# Vγ1 γδ T cells steer airway macrophages toward a profibrotic response in an autochthonous lung cancer mouse model

**DOI:** 10.1126/sciadv.adu8802

**Published:** 2026-03-04

**Authors:** Ximena L. Raffo-Iraolagoitia, Amanda J. McFarlane, Sarah Laing, Ryan Corbyn, Lindsey W. G. Arnott, Frédéric Fercoq, Lynn McGarry, Judith Secklehner, Marco De Donatis, John B. G. Mackey, Bjorn Kruspig, Robert Wiesheu, Ya-Ching Hsieh, Robin Shaw, Kai Rakovic, John Le Quesne, Graeme Clark, Colin Nixon, Crispin Miller, Kristina Kirschner, Calum C. Bain, Daniel J. Murphy, Seth B. Coffelt, Leo M. Carlin

**Affiliations:** ^1^ Cancer Research UK Scotland Institute, Glasgow, UK.; ^2^ School of Cancer Sciences, University of Glasgow, Glasgow, UK.; ^3^ Inflammation, Repair & Development, Imperial College London, London, UK.; ^4^ Department of Histopathology, NHS Greater Glasgow and Clyde, Glasgow, UK.; ^5^ Centre for Immunobiology, School of Infection and Immunity, University of Glasgow, Glasgow, UK.; ^6^ Institute for Regeneration and Repair, University of Edinburgh, Edinburgh, UK.

## Abstract

γδ T cells are important for host defense at the respiratory mucosa, acting directly or through interactions with other cells. However, how γδ T cells influence other immune cells in the lung remains unclear. Using a genetically engineered mouse model of lung cancer, we show that tumors drive expansion of both CD27^+^ and CD27^−^ γδ T cells. Advanced microscopy techniques indicated that CD27^−^ γδ T cells are enriched in tumors, whereas CD27^+^ γδ T cells are more prone to interact with macrophages in tumor-associated adventitial cuffs. SiglecF^low^ profibrotic airway macrophages were more prevalent in lung tumor-bearing mice than tumor-free mice. This profibrotic subset was reduced in lungs when the cancer model was crossed to *Tcrd* knockout mice or treated with Vγ1-depleting antibodies but not in *TcrgV4/6* knockout mice. Thus, our findings implicate Vγ1 γδ T cells in driving tumor-associated airway macrophage functional imprinting. Determining the translatability to human health may offer new avenues for refining patient management and immunotherapeutic strategies.

## Teaser

In lung cancer, airway macrophages are modulated by an unconventional T cell subset, relevant to care for pulmonary comorbidities.

## INTRODUCTION

Pulmonary homeostasis relies on intricate cellular networks to secure gas exchange. In the lung, the immune response balances a high threshold for innocuous particles and robust clearance of invading pathogens through an interplay between pulmonary epithelial cells, tissue-resident immune cells, and leukocytes recruited from circulation (*1*). Adding another layer of complexity, these cells are segregated into microanatomically distinct compartments such as parenchyma (including the respiratory bronchioles, alveolar ducts, and alveoli), perivascular adventitial cuffs (extracellular matrix-rich hubs for regional immune responses), and the intravascular leukocyte pool contained by the endothelium (*2*, *3*). Comprehensively studying the changes that arise in these networks when epithelial cells become malignant might be the key to uncovering new targets for immunotherapies in lung cancer.

Gamma delta (γδ) T cells are unconventional T cells that provide protective and/or immunomodulatory functions at many barrier sites, including the lung. With a limited T cell receptor diversity, γδ Τ cells using Vγ1, Vγ4, or Vγ6 gamma chains have been implicated in lung physiology and pathology in mice (*4*, *5*). In patients with non–small cell lung cancer (NSCLC), the presence of Vδ1 T cells is associated with improved survival (*6*). In mice, γδ Τ cells consist of several subsets that express a variety of cytokines and other mediators of the immune response and are generally subdivided into two populations depending on the expression of the cell surface marker CD27 (*5*). In cancer, CD27^+^ γδ T cells are thought to play an antitumoral role due to their production of interferon-γ (IFN-γ) and cytotoxic ability (*7*), contrasting with their CD27^−^ counterparts which produce interleukin-17 (IL-17), supporting tumor development through a neutrophil-mediated mechanism (*4*, *8*, *9*). However, our current understanding of their role in lung cancer is limited because their precise location within the pulmonary topology remains unknown.

Resting pulmonary γδ Τ cells have been shown to interact preferentially with myeloid cells, including macrophages (*10*). In the steady-state lung, the macrophage compartment is heterogeneous, comprising alveolar macrophages (AMs) and interstitial macrophages (IMs), which are transcriptionally and developmentally distinct. AMs are thought to maintain themselves autonomously in health through in situ self-renewal; however, upon lung insult, monocytes can also give rise to AMs (mo-AMs) (*11*). It has been proposed that, due to their “monocyte legacy,” they display increased plasticity and reactivity, changing their function in response to environmental cues, thus influencing the outcome of lung infections, lung cancer, and chronic inflammatory disease (*12*, *13*). In NSCLC, AMs promote tumor formation at early stages (*14*), and it has been proposed that these cells are replaced by mo-AMs, which maintain an immunosuppressive role opposing antitumor immunity (*15*). There have been several clinical trials attempting to target macrophages in NSCLC such as pexidartinib (NCT02452424), ARRY-382 (NCT02880371), emactuzumab (NCT02323191), LY3022855 (*16*, *17*), and AMG 80 (NCT02713529) (*18*, *19*); however, no clinical benefit has been proven. In a mouse model of lung cancer, it was shown that AMs promote the expansion of γδ Τ cells (*9*). Whether, conversely, γδ Τ cells play a role in regulating AMs has not yet been explored.

Here, in the context of lung adenocarcinoma (LuAd), we report that γδ T cells shape the reactivity of tumor-associated AMs and interact via the γδ-TCR. γδ T cells promote the accumulation of AMs with profibrotic features and a signature associated with poor outcomes in patients with LuAd. In the absence of γδ T cells, AMs produce more inflammatory cytokines and exhibit increased phagocytosis. Our findings may have implications not only for the design of immunotherapies targeting tumor-associated AMs but also for improving patient care for LuAD comorbidities.

## RESULTS

### Pulmonary γδ Τ cells increase in lung cancer

**Fig. 1.**
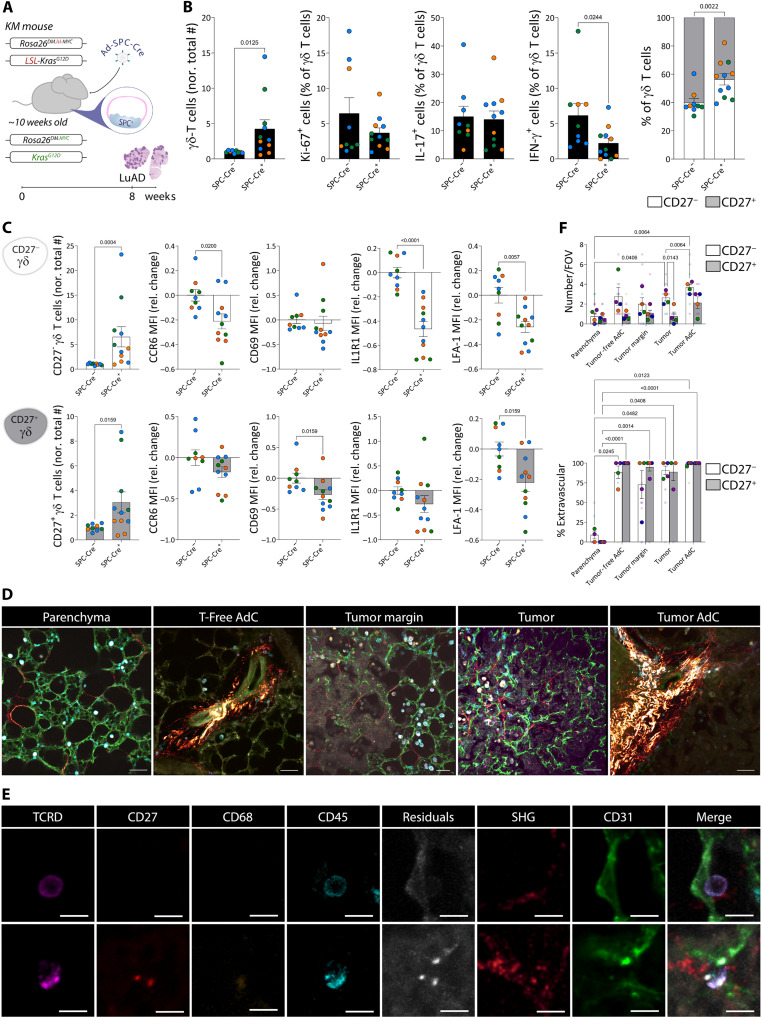
Pulmonary γδ Τ cells are increased, and change phenotype, in a GEMM of LuAd. (**A**) SPC-Cre^+^ KM mouse model of LuAd. Eight weeks after transgene activation, lungs were harvested for flow cytometry (B and C) or confocal microscopy (D to F). (**B**) Left: Total numbers of γδ Τ cells normalized to SPC-Cre^−^ KM controls. Right: Percentage of positive cells for Ki-67, IL-17, IFN-γ, and CD27. (**C**) Gated on CD27^−^ (white bars) and CD27^+^ (gray bars) γδ Τ cells, total numbers of cells normalized to SPC-Cre^−^ KM controls, and MFI relative change for CCR6, CD69, IL1R1, and LFA-1. Each dot represents a mouse, colored by independent experiment. (**D**) Representative fields of view for each region type in PCLSs from SPC-Cre^+^ KM mice; vasculature (CD31, green), collagen (second harmonic generation, glow palette), spectral unmixing residuals (gray), leukocytes (CD45, cyan), macrophages (CD68, orange), CD27 (red), and TCRδ (magenta). Scale bars, 50 μm. (**E**) Individual and merged channels for a representative CD27^−^ (top) and CD27^+^ (bottom) γδ Τ cell. Scale bars, 10 μm. (**F**) Quantification of CD27^−^ and CD27^+^ γδ Τ cell mapping. Each color represents a mouse, small dots for fields of view (FOV) and big dots for means. Data are presented as means ± SEM. Data were analyzed by the Mann-Whitney test (*n* = 9 to 11 mice per group) (B and C); two-way ANOVA followed by Tukey’s multiple comparisons posttest (*n* = 4) (F).

To characterize the response of γδ Τ cells to lung cancer, we used a well-established autochthonous genetically engineered mouse model (GEMM). Here, a heterozygous point mutation in the endogenous *Kras* locus (*Kras^G12D^*) and modest overexpression of the oncogene *MYC* from the homozygous *Rosa26* locus were induced by intranasal delivery of an adenoviral vector containing Cre recombinase under the control of the surfactant protein C (*Spc*) promoter (herein referred to as SPC-Cre^+^ KM or tumor-bearing mice) (*20*). Eight weeks after Cre delivery, we analyzed spleen, blood, and lungs by flow cytometry (Fig. 1A and Table 1). At this time point, mice showed no clinical signs of disease by physical examination, but early-stage LuAds were evident in the lungs of SPC-Cre^+^ KM mice by histology (fig. S1A). This was accompanied by an increase in lung leukocytes (fig. S1B) and reshaping of the immune landscape with augmented monocytes, IMs, and AMs (fig. S1C), specific to tumor-bearing mice, as SPC-Cre^+^ mice without the KM alleles or only *Kras^G12D^* showed no changes (fig. S1B). Although γδ Τ cells remained a minority among other leukocytes (fig. S1D), they were increased more than fourfold in tumor-bearing mice (Fig. 1B). However, we did not find evidence of increased in situ γδ Τ cell proliferation by staining for Ki-67 or their production of IL-17 or IFN-γ in tumor-bearing mice after ex vivo stimulation; IFN-γ was reduced (Fig. 1B). Accordingly, we found a relative decrease in the ratio of CD27^+^ subpopulation over their CD27^−^ counterparts, compared with tumor-free mice (Fig. 1B), despite both subsets being increased (Fig. 1C). We next measured the expression of surface markers required for migration, activation, and adhesion in both subsets relative to their equivalents in tumor-free mice (Fig. 1C and fig. S1E). CCR6, whose down-regulation is required for recruitment of γδ Τ cells to inflamed tissues (*21*), and IL1R1, critical for IL-17 production (*22*), were reduced in CD27^−^ but not in CD27^+^ γδ Τ cells of tumor-bearing lungs. Conversely, the activation marker CD69 was reduced in CD27^+^ but not in CD27^−^ γδ Τ cells of tumor-bearing lungs. Both γδ Τ cell subsets had reduced expression of LFA-1, an adhesion molecule involved in the formation of immune synapses that enhances cytotoxicity (*23*) in tumor-bearing lungs. Although small, none of these changes were mirrored in peripheral blood γδ Τ cells (fig. S1F), suggesting that the two subsets are regulated locally in the tumor microenvironment (TME).

**Table 1.** Flow cytometry reagents.

Fluorescent dye	Clone	Company	Panel					
			Lymphoid	Myeloid	IC	γδ	Mac sort	AM and γδ sort
CCR2 BV785	SA203G11	BioLegend		x			x	
CCR6 BV785	29-2 L17	BioLegend			x			
CD115 BV421	AFS98	BioLegend		x			x	
CD11b APC-eFluor780	M1/70	Invitrogen				x		
CD11b BV650	M1/70	BioLegend	x	x			x	x
CD11c AF700	N418	BioLegend						x
CD11c BV510	N418	BioLegend		x			x	
CD169 PE	3D6.112	BioLegend		x			x	
CD19 APC-eFluor780	1D3	Invitrogen				x		
CD19 BV421	6D5	BioLegend						x
CD19 BV605	6D5	BioLegend	x					
CD27 BV510	LG.3A10	BioLegend	x			x		x
CD27 PE-Dazzle 594	LG.3A10	BioLegend			x			
CD3 BV650	17A2	BioLegend			x	x		
CD3 PerCP-Cy5.5	145-2C11	BioLegend	x					x
CD4 BV605	GK1.5	BioLegend			x	x		
CD4 PE-Cy7	RM4-4	BioLegend	x					
CD44 APC	IM7	BioLegend	x					
CD44 PerCP-Cy5.5	IM7	BioLegend				x		
CD45 BV711	30-F11	BioLegend	x	x	x	x	x	x
CD62L BUV395	MEL-14	BioLegend				x		
CD62L BV785	MEL-14	BioLegend	x					
CD64 PE-CY7	X54-5/7.1	BioLegend		x			x	
CD69 BV510	H1.2F3	BioLegend			x			
CD8 BUV395	53-6.7	BioLegend	x		x			
CD8 BUV805	53-6.7	BD Biosciences				x		
CX3CR1 BV605	SA011F11	BioLegend		x			x	
DAPI		Thermo Fisher Scientific						x
EpCam APC-eFluor780	G8.8	Invitrogen				x		
EpCam BV421	G8.8	BioLegend						x
F4/80 FITC	A3-1	Bio-Rad		x			x	
FOX3P PE	MF-14	BioLegend			x			
IA/IE PE-Dazzle 594	M5/114.15.2	BioLegend		x			x	
ICOS PE	7E.17G9	BioLegend	x					
IFN-γ APC-Cy7	XMG1.2	BioLegend			x			
IL-17a PE-Cy7	eBio17B7	Invitrogen			x			
IL1R1 AF647	35F5	BD Biosciences			x			
Ki-67 AF700	16A8	BioLegend			x			
LFA-1 PerCP-Cy5.5	M17/4	BioLegend			x			
Ly6C PE-Cy7	HK1.4	BioLegend				x		
Ly6C PerCP-Cy5.5	HK1.4	BioLegend		x			x	
Ly6G APC-Cy7	1A8	BioLegend					x	
Ly6G BUV395	1A8	BD Biosciences		x				
Ly6G BV421	1A8	BioLegend						x
NKp46 BV421	29A1.4	BioLegend	x		x			
SiglecF AF647	E50-2440	BD Biosciences		x			x	x
TCRD FITC	GL3	BioLegend	x		x	x		x
Vγ1 BV605	2.11	BD Biosciences						x
Vγ1 PE	2.11	BioLegend				x		
Vγ4 APC	UC3-10A6	BioLegend				x		
Vγ6 PB	1C10-1F7	Hybridoma, in-house				x		
Zombie NIR		BioLegend	x	x		x	x	
Zombie Yellow		BioLegend			x			

Next, we mapped the distribution of γδ Τ cells within different topological compartments present in LuAd by confocal microscopy. We imaged five distinct regions on the basis of the presence or absence of tumors and fibrillar collagen-rich adventitial cuffs (visualized by second harmonic generation), namely, parenchyma, tumor-free adventitial cuffs, tumor margin, tumor, and tumor-associated adventitial cuffs (Fig. 1D). In each region, we quantified γδ Τ cells as CD45^+^CD68^−^TCRδ^+^ and segregated these cells as either CD27^−^ or CD27^+^ (Fig. 1E), determining their position relative to the vasculature (CD31). Whereas CD27^−^ γδ Τ cells were enriched in tumor regions, particularly surrounding adventitial cuffs, CD27^+^ γδ Τ cells did not show a notable tropism for a location. Both subsets were found in blood vessels in the parenchyma; however, they mostly infiltrated the tissue in the other regions (Fig. 1F and movies S1 and S2). Together, the lack of in situ proliferation (by Ki-67) and the extravascular localization revealed by confocal imaging suggest that γδ Τ cells are recruited to tumors. They nevertheless exhibit unchanged IL-17 production, reduced IFN-γ production, and diminished cell surface activation markers.

### γδ Τ cells interact with tumor-associated macrophages

**Fig. 2.**
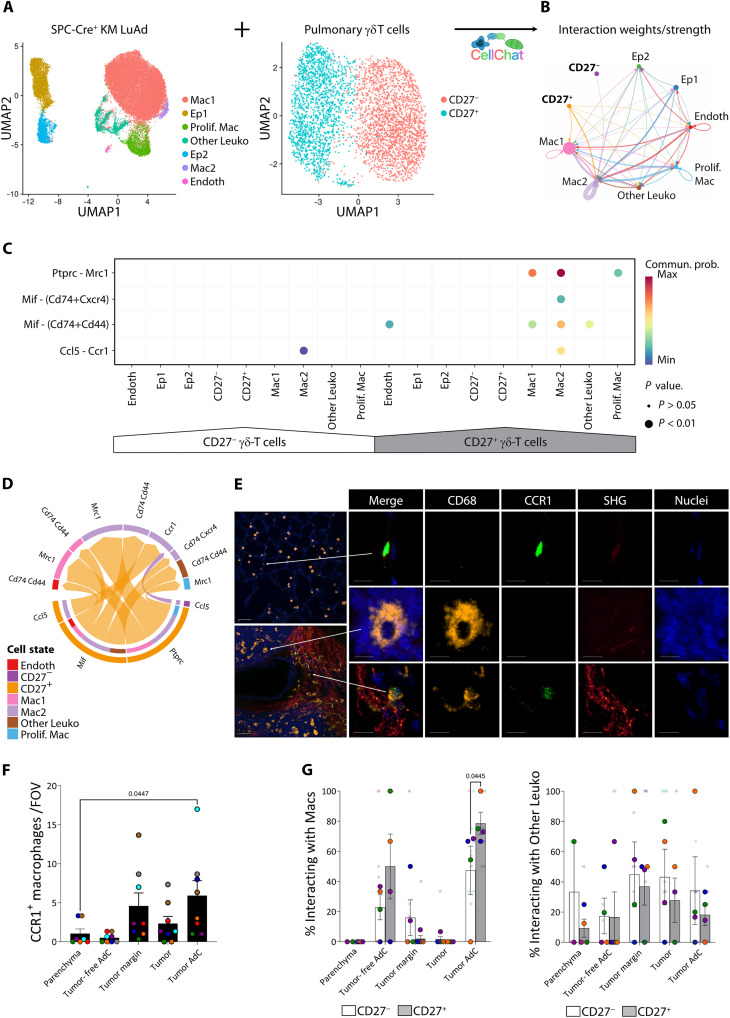
γδ Τ cells interact with TAMs. (**A**) ScRNA-seq data from microdissected SPC-Cre^+^ KM mouse lungs tumors and naïve pulmonary γδ Τ cells (*4*) were merged for CellChat analysis. (**B**) Aggregated cell-cell communication network visualized in a circle plot showing the total interaction weights/strength between any two clusters, with edge weights proportional to the interaction strength and circle sizes proportional to the number of cells in each cell group. (**C**) Bubble plot showing all significant interactions from γδ Τ cells. (**D**) Chord diagram showing all significant interactions from γδ Τ cells. (**E**) Left: Representative fields of view for parenchyma (top) and tumor-associated adventitial cuff (bottom) in PCLSs from SPC-Cre^+^ KM;*iCcr*-*rep*^+/+^ mice; macrophages (CD68, orange), CCR1 (Clover, green), collagen (second harmonic generation, glow palette), and nuclei (SYTOX Blue, blue). Scale bars, 50 μm. Right: Individual and merged channels for a representative CCR1^+^CD68^−^ (top), CCR1^−^CD68^+^ (middle), and CCR1^+^CD68^+^ (bottom) cell. Scale bars, 10 μm. (**F** and **G**) Quantification by confocal microscopy of (F) CCR1^+^ macrophages mapping in different topologies in LuAd. Each color represents a mouse, dots for the mean of one to three fields of view per mouse (*n* = 7 to 8). (G) CD27^−^ (white) and CD27^+^ (gray) γδ Τ cells interacting with macrophages (Macs; CD45^+^CD68^+^; left) and other leukocytes (CD45^+^CD68^−^; right) grouped by different topologies in LuAd. Each color represents a mouse, small dots for fields of view and big dots for means (*n* = 4). Data are presented as means ± SEM (F and G). Data were analyzed by the Kruskal-Wallis test followed by Dunn’s multiple comparisons test (F) and mixed-effects restricted maximum likelihood followed by uncorrected Fisher’s least significant difference (LSD) tests (G).

The interaction of γδ Τ cells with other cells in the lung is poorly understood. Therefore, we sought to explore this network using CellChat (*24*). We performed total cell single-cell RNA sequencing (scRNA-seq) of tumors microdissected under a stereomicroscope from SPC-Cre^+^ KM mouse lungs (GSE307621). Following the standard Seurat pipeline (*25*), we found seven cell clusters comprising epithelial cells, endothelial cells, and leukocytes (Fig. 2A and fig. S2A). Of note, we could not identify a cluster of γδ Τ cells, even at higher resolutions, although this is not entirely unexpected as γδ Τ cells in scRNA-seq experiments often do not cluster distinctly and appear embedded among more abundant lymphocytes such as CD8^+^ T cells and natural killer (NK) cells (*26*). To predict interactions that steady-state γδ Τ cells might have once recruited to the TME, we then leveraged these data with our scRNA-seq data from sorted pulmonary γδ Τ cells (*4*). This dataset solely contained two clusters corresponding to CD27^−^ and CD27^+^ γδ T cells (Fig. 2A and fig. S2B). Cellular communication modeling revealed all the interactions computed in the CellChat database that could be involved between the nine clusters together (Fig. 2B). Looking in detail at only those involving γδ T cells, we noticed that both CD27^−^ and CD27^+^ clusters were acting exclusively as “senders” in the significant pathways identified. The most probable “receivers” of outgoing signaling patterns from γδ T cells, particularly among those from the CD27^+^ subset, were macrophages through *Mrc1* (encoding CD206, a marker frequently up-regulated in protumor macrophages). Other putative receptor-ligand pairs included *Mif/Cd74* and *Ccl5/Ccr1* (Fig. 2C). In a chord diagram, using both subsets of γδ T cells as sources (bottom part of the ring), it was clear that most of the signaling outputs reaching macrophages (pink, lilac, and blue) were derived from CD27^+^ γδ T cells (orange arrows), and a minority of the *Ccl5* was supplied by CD27^−^ γδ T cells (violet arrow) (Fig. 2D). Only a small fraction of the total interactions detected was received by clusters other than macrophages. Of note, the absence of the γδ-TCR among the hits does not exclude its involvement as many of its ligands remain largely unknown.

Of the predicted receptors, CD206 and CD74 are widely expressed on lung macrophages. Thus, we focused on CCR1, whose expression on macrophages in lung cancer has not yet been determined but increases on macrophages during pulmonary metastasis progression and whose inhibition reduces pulmonary metastatic burden (*27*). We crossed KM mice with *iCcr*-*rep*^+/+^ mice, which carry a bacterial artificial chromosome containing the inflammatory *Ccr* gene cluster with *Ccr1* replaced by *Clover* (*28*), to locate the CCR1^+^ macrophages within the different lung topologies of tumor-bearing mice. In each region, we quantified cell-compatible CD68^+^CCR1^+^ spots, excluding CD68^+^CCR1^−^ and CD68^−^CCR1^+^ spots, using Imaris image analysis software (Fig. 2E). Compared to parenchyma regions, CCR1^+^ macrophages were enriched in tumor-associated adventitial cuffs (Fig. 2F). Together, these results allowed us to hypothesize that γδ T cells communicate with tumor-associated macrophages (TAMs).

To address this hypothesis, we quantified the interactions of CD27^+^ γδ Τ cells and CD27^−^ γδ Τ cells with macrophages (CD45^+^CD68^+^) and other leukocytes (CD45^+^CD68^−^) within different topologies in LuAd (Fig. 2G). We used a semiautomated approach in Imaris image analysis software, manually verifying that cells were in contact and that there were no other structures, such as endothelia, between them (movie S3). As predicted, a higher percentage of CD27^+^ than CD27^−^ γδ Τ cells was in close contact with macrophages, although only in tumor-associated adventitial cuffs. Moreover, in this location, most of the CD27^−^ γδ Τ cells were in immediate proximity with macrophages. Interactions with leukocytes other than macrophages were also observed in the different topologies without showing any tropism or bias toward either subset of γδ Τ cells, although this broad category could mask effects on particular cell types. As the quantification was performed in fixed tissue and could not discriminate whether the cells were dynamically interacting or just transient bystanders, we next explored live precision-cut lung slices (PCLSs) to capture the behavior of γδ Τ cells and TAMs in close contact. In this way, we provided evidence supporting the occurrence of prolonged interactions of γδ Τ cells with TAMs in the TME (movie S4).

Consistent with CellChat predictions, confocal imaging placed TAMs and γδ Τ cells together, particularly the CD27^+^ subset, within tumor-associated adventitial cuffs. Notably, these regions were enriched in CCR1^+^ macrophages, one of the predicted interaction molecules, providing spatial and molecular evidence supporting an interplay between γδ Τ cells and TAMs in LuAd.

### A subset of tumor-associated AMs displays a profibrotic signature

**Fig. 3.**
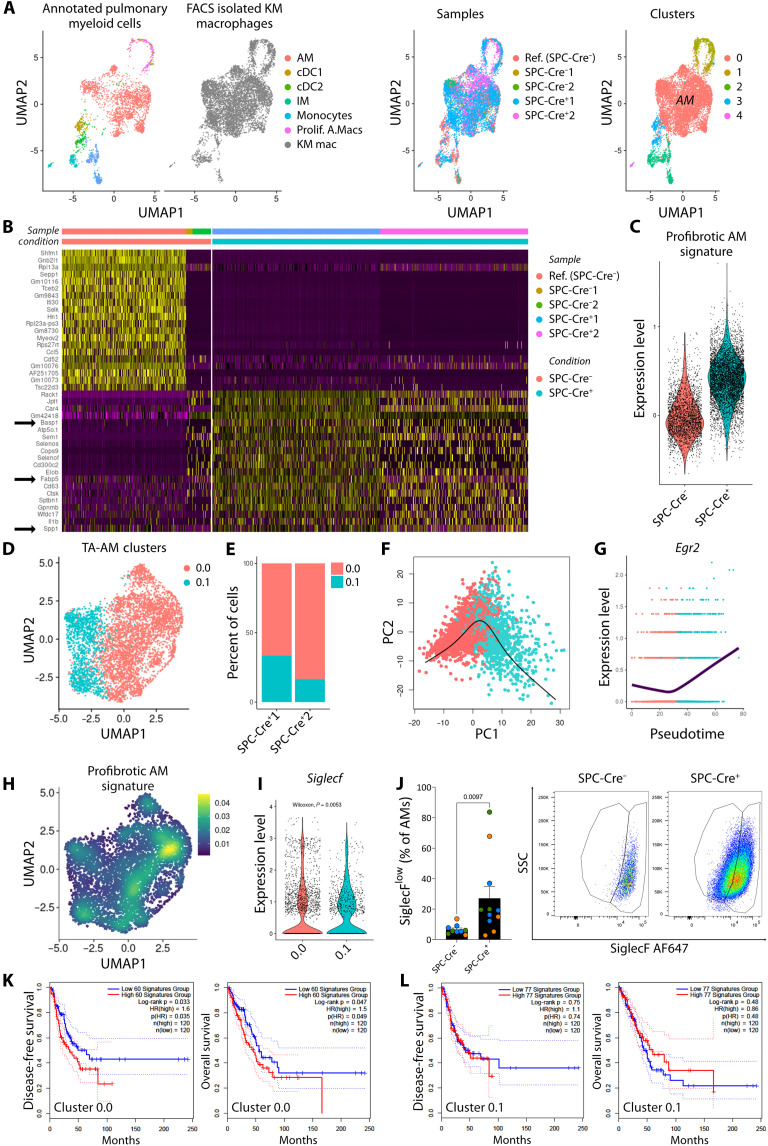
A subset of tumor-associated AMs displays a profibrotic signature. (**A**) Uniform Manifold Approximation and Projection (UMAP) plots of scRNA-seq from a reference pulmonary myeloid cell dataset (*29*) integrated with FACS-isolated macrophages from microdissected KM lungs (SPC-Cre^−^ and SPC-Cre^+^, *n* = 2 mice per group) colored by annotation (left), sample (center), and cluster (right). (**B**) Heatmap showing the top 20 most DEGs in AMs (cluster 0) across conditions (SPC-Cre^−^ in red versus SPC-Cre^+^ KM mice in blue). (**C**) Violin plot showing the expression of a consensus profibrotic AM signature (*30*) by condition. AMs from SPC-Cre^+^ KM mice (TA-AM) were subset and reclustered for further analysis (D to I). (**D**) UMAP plot of two subclusters of TA-AMs. (**E**) Stacked plot showing the percentage of each subcluster in two independent experiments (*n* = 2). (**F**) Principal curve describing the global lineage structure with cluster 0.0 as starting point using Slingshot (*31*) for pseudotime inference. (**G**) Expression level of *Egr2* over pseudotime from cluster 0.0 to cluster 0.1. (**H**) Density plot according to the profibrotic AM signature on UMAP dimensionality reduction for TA-AMs. (**I**) Violin plot showing the expression of *Siglecf* in TA-AMs clusters, analyzed by the Wilcoxon test. (**J**) Percentage of SiglecF^low^ cells gated on AMs by flow cytometry. Each dot represents a mouse, colored by experiment (*n* = 9 to 11 mice per group). Data are presented as means ± SEM. Data were analyzed by the Mann-Whitney test. (**K** and **L**) Disease-free and overall survival based on the expression of the signatures of cluster 0.0 (K) or cluster 0.1 (L) for patients with LuAd in the GEPIA2 server, hazards ratio (HR) based on the Cox PH Model, and log-rank test (*32*).

TAMs are thought to promote tumor growth by a plethora of mechanisms, increasing the sources of heterogeneity of the different subsets of macrophages residing in the lungs. Using scRNA-seq of microdissected lung tumors, we found three clusters with transcriptional features of macrophages, including *Ear1*, *Car4*, and *Mrc1* (Fig. 2A and fig. S2C). To address the heterogeneity of this population, we performed scRNA-seq of fluorescence-activated cell sorting (FACS)–isolated CD64^+^F4/80^+^ cells, including both AMs (SiglecF^+^CD11b^−^) and IMs (CD64^+^CD11b^+^), from tumor-free lungs (SPC-Cre^−^ KM) and from tumors microdissected under a stereomicroscope from SPC-Cre^+^ KM lungs (fig. S3A). As most of the cells recovered were from tumor-bearing mice, we integrated our data with a reference dataset (*29*) of pulmonary myeloid cells from control mice to enhance the cell numbers from SPC-Cre^−^ lungs. Considering the annotation of the reference dataset, we identified the predominant cluster in our data as AMs (Fig. 3A). In addition, we found proliferating AMs and IMs, as well as dendritic cells and monocytes, but all these cells were scarce. We then built a heatmap for the AMs (cluster 0) with the 20 most differentially expressed genes (DEGs) across our conditions (tumor-free SPC-Cre^−^ versus tumor-bearing SPC-Cre^+^ KM) (Fig. 3B). We noticed that three genes (*Basp1*, *Fabp5*, and *Spp1*) of the 20 markers were part of a consensus profibrotic signature driven by mo-AMs (*30*). Tumor-associated AMs were enriched for this signature (Fig. 3C and fig. S3B), suggesting that they could be comprised, in part, of profibrotic mo-AMs.

Next, we reclustered tumor-associated AMs, revealing two transcriptionally distinct subpopulations with differences in 46 of the 50 Gene Set Enrichment Analysis (GSEA) Hallmark gene sets, with cluster 0.1 enriched in immunoreactive pathways (Fig. 3D and fig. S3C, see arrows). These two clusters were similarly represented in both independent experiments (Fig. 3E). To explore a developmental difference between the clusters, we used Slingshot (*31*) to fit a smooth trajectory using principal curves to describe the underlying lineage (Fig. 3F). When we analyzed the expression of *Egr2*, a determinant of AM identity and function (*29*), we observed that the expression of this transcription factor increased gradually over pseudotime from cluster 0.0 toward cluster 0.1 (Fig. 3G). The profibrotic mo-AM signature reported by Joshi *et al.* (*30*) was more prevalent in cluster 0.0 (Fig. 3H). In search for a surface marker to distinguish these clusters by flow cytometry, we examined the expression of *Siglec5*, which encodes SiglecF and is considered the de facto marker of fully mature AMs. This marker was expressed at significantly lower levels by tumor-associated AMs belonging to cluster 0.0 (Fig. 3I). We detected, by flow cytometry, the enrichment of a subpopulation of SiglecF^low^ AMs in tumor-bearing lungs (Fig. 3J) previously described in models of acute lung injury where mo-AMs repopulate the lung after bleomycin treatment (*11*) and influenza infection (*12*). To validate the correspondence between our gated SiglecF^low^ AMs and cluster 0.0, we performed bulk RNA sequencing (RNA-seq) on FACS-isolated SiglecF^low^ and SiglecF^hi^ AMs from tumor-bearing lungs and scored their DEGs in the scRNA-seq dataset. Accordingly, the module score for DEGs up-regulated in SiglecF^low^ AMs was higher in cluster 0.0, and the equivalent for SiglecF^hi^ AMs was higher in cluster 0.1. Moreover, the enriched gene ontology terms based on significantly up-regulated genes in SiglecF^low^ AMs included extracellular matrix, structural constituent and binding, and collagen trimer (fig. S3D). Together, these findings reveal that tumor-associated AMs are transcriptomically composed of two subsets: a more abundant one with a profibrotic profile and a minor one displaying an immunoreactive profile.

To explore the potential relevance to human health, we performed a survival analysis based on the signature of each cluster using the human orthologs of all differentially overexpressed genes in GEPIA2 (*32*). Whereas patients with LuAd and high expression of the cluster 0.0 signature showed reduced disease-free and overall survival (Fig. 3K), no significant stratification was observed when we used the cluster 0.1 signature (Fig. 3L). This finding complements previous research in mice using intravenously injected cancer cells, where mo-AMs were associated with enhanced tumor spread (*33*). As the signature of the profibrotic cluster correlates with poor prognosis in LuAd, but the immunoreactive one does not, understanding what regulates the abundance of these subsets could be relevant for the care and clinical management of patients with LuAd.

### γδ Τ cells influence AM function, and TCRD clusters during their interaction

**Fig. 4.**
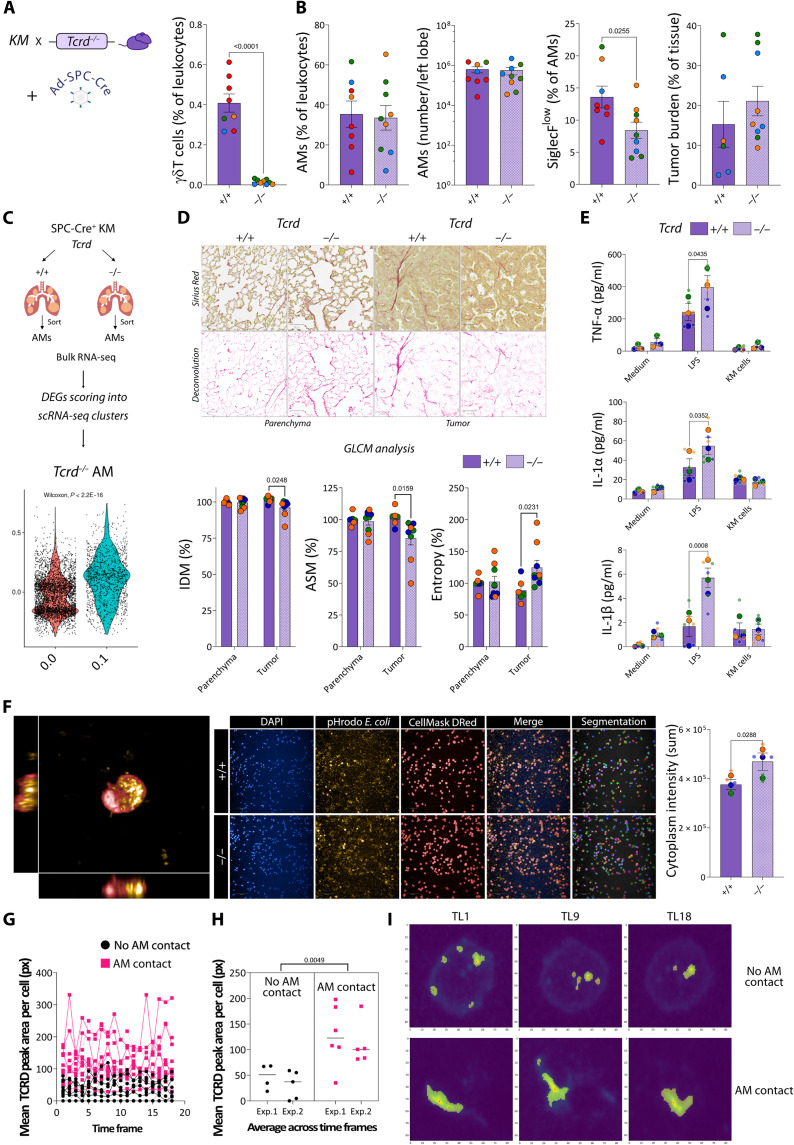
γδ Τ cells influence AM function and TCRD clusters during their interaction. Lungs from KM;*Tcrd*^+/+^ and KM;*Tcrd*^−/−^ mice 8 weeks postinduction (A to F). (**A**) γδ Τ cells (% of leukocytes). (**B**) AMs (% of leukocytes), total number/left lobe, and SiglecF^low^ cells (% of AMs), flow cytometry (*n* = 8 to 9 mice per group). Tumor-burden whole lungs, histology (*n* = 6 to 9 mice per group). (**C**) Violin plots of module scores per cell TA-AM clusters 0.0 and 0.1, DEGs in bulk RNA-seq of sorted AMs (*n* = 3 mice per group). (**D**) GLCM analysis, whole lung Sirius Red. Scale bars, 50 μm. IDM, ASM, and entropy, parenchyma, and tumor (*n* = 6 to 9 mice per group). (**E**) Sorted AM ex vivo TNF-α, IL-1α, and IL-1β production 24 hours with culture medium, LPS, or KM cells (*n* = 3 mice per group). (**F**) AM *pHrodo E. coli* uptake (left, 63x). Representative fields of AMs from SPC-Cre^+^ KM;*Tcrd*^+/+^ lungs (top) and KM;*Tcrd*^−/−^ lungs (bottom) (20x). AM *pHrodo E. coli* cytoplasm intensity. Each dot represents a mouse, colored by experiment (*n* = 3 mice per group). Smaller dots (E and F) duplicate cultures. Data means ± SEM, *t* test if Gaussian distribution (A, B, and F), Wilcoxon test for cluster 0 (3698 cells) versus cluster 1 (1282 cells) (*n* = 2 mice) (C), two-way ANOVA with Sidak post hoc (D and E). Quantification TCRD clustering in live PCLSs from SPC-Cre^+^ KM mice (G to I). (**G**) Mean TCRD peak area per cell for each time point; solid and dotted lines indicate independent experiments. No AM contact: 9 cells (4 to 5 per experiment). AM contact: 11 cells (5 to 6 per experiment). (**H**) Average over time of the mean TCRD peak area per cell, by experiment. Each dot represents a γδ Τ cell, data as mean for each experiment and condition. Paired *t* test of experiment-level means (*n* = 2 mice). (**I**) Representative TCRD masks and peaks for a γδ Τ cell in the absence (top) or presence (bottom) of AM contact at time points 1, 9, and 18.

Given the physical interaction between CD27^+^ γδ Τ cells and TAMs, and the accumulation of SiglecF^low^ AMs in the lungs of tumor-bearing mice with a profibrotic signature, we determined how macrophages, and specifically SiglecF^low^ AMs, are affected by the absence of γδ Τ cells. Thus, we crossed *Tcrd*^−/−^ mice with KM mice to generate cohorts of KM;*Tcrd*^+/+^ and KM;*Tcrd*^−/−^ mice. Eight weeks after allele activation with SPC-Cre, we analyzed tumor-bearing lungs as before (Fig. 4A). Whereas AMs were not affected in proportion or total number by the absence of γδ Τ cells, we observed that tumor-bearing *Tcrd*^−/−^ mice had a decreased proportion of SiglecF^low^ AMs compared with tumor-bearing *Tcrd*^+/+^ mice. The change observed in the percentage of SiglecF^low^ AMs was independent of differences in tumor burden as these were equivalent at this time point (Fig. 4B).

As the mechanisms by which AMs acquire either profibrotic or immunoreactive profiles after lung insult are largely unknown (*13*), we sought to explore whether γδ Τ cells play a role in determining these AM traits. Using bulk RNA-seq of sorted AMs, we found that the module score for DEGs up-regulated in AMs from tumor-bearing *Tcrd*^−/−^ mice was lower for cluster 0.0 (Fig. 4C). This suggests that, in the absence of γδ Τ cells, AMs from tumor-bearing mice have a less “profibrotic” transcriptional profile. Furthermore, the Hallmark gene sets for the AMs from tumor-bearing *Tcrd*^−/−^ mice were markedly consistent with an “immunoreactive” transcriptome, including tumor necrosis factor–α (TNF-α), IFN-γ, IL-6, IFN-α, and inflammatory responses/signaling (fig. S3E). Therefore, we quantitatively compared the fibrillar collagen texture in lungs from *Tcrd*^−/−^ and *Tcrd*^+/+^ tumor-bearing mice by gray-level co-occurrence matrix (GLCM) analysis of Sirius Red staining (*34*). Although no changes were observed in parenchyma, we found significant differences in tumors. In *Tcrd*^−/−^ mice, we observed a reduction in inverse difference moment and angular second moment, reflecting increased differences in local intensity between neighboring pixels and decreased uniformity in the collagen texture, respectively. Concurrently, entropy was increased, indicating a less organized collagen texture (Fig. 4D) (*35*). Together, these changes suggest that the absence of γδ Τ cells had a local impact on extracellular matrix remodeling, which may contribute to a less densely and less uniformly structured collagen deposition.

To evaluate the functional effect of γδ Τ cells on AMs in LuAd, we isolated AMs by FACS from *Tcrd*^−/−^ and *Tcrd*^+/+^ tumor-bearing mice and probed their cytokine production and phagocytosis ex vivo. Whereas there were no differences in unstimulated AMs, or those cocultured with a KM tumor-derived cell line, upon stimulation with lipopolysaccharide (LPS), AMs from *Tcrd*^−/−^ tumor-bearing mice secreted more TNF-α, IL-1α, and IL-1β than those from their γδ Τ cell–sufficient counterparts (Fig. 4E), consistent with our bulk RNA-seq data. After coculture with *pHrodo Escherichia coli*, we quantified the cytoplasm intensity for the fluorescent bioparticles, and AMs from *Tcrd*^−/−^ tumor-bearing lungs also had evidence of increased phagocytosis (Fig. 4F). Overall, we found that γδ Τ cells are key determinants of the reactivity of AMs in tumor-bearing mice. In their absence, AMs develop immunoreactive traits, producing more pro-inflammatory cytokines and exhibiting increased phagocytosis.

Having previously observed prolonged interactions reminiscent of an immunological synapse (movie S4), we next examined the spatial organization of TCRD on γδ Τ cells with or without AM contact to explore potential mechanisms underlying these functional effects. To this end, we performed live PCLS imaging from three lung lobes of tumor-bearing mice. Across two independent experiments, we captured 20 γδ Τ cells in total: 9 in the absence of AM contact (movie S5) and 11 in the presence of AM contact (movie S6). For each time frame, we cropped the TCRD channel to a fixed area encompassing a complete γδ Τ cell, ensuring that all fluorescence intensity measured originated from that cell. We then quantified the surface of local maxima, number of peaks, and the mean TCRD peak area per cell (Fig. 4G and fig. S4). Averaging this parameter across all time frames revealed a consistent increase in TCRD cluster size (mean TCRD peak area per cell) in both independent experiments (mean difference: 79.92 px; range: 79.31 to 80.53 px) (Fig. 4, H and I). Together, these findings implicate γδ Τ cells as “switches” controlling AM cell functional programs in tumor-bearing mice and point to TCRD clustering during their interaction as a potential mechanism driving this effect.

### Vγ1^+^ γδ Τ cells contribute to SiglecF^low^ AM accumulation in a CSF1-independent manner

**Fig. 5.**
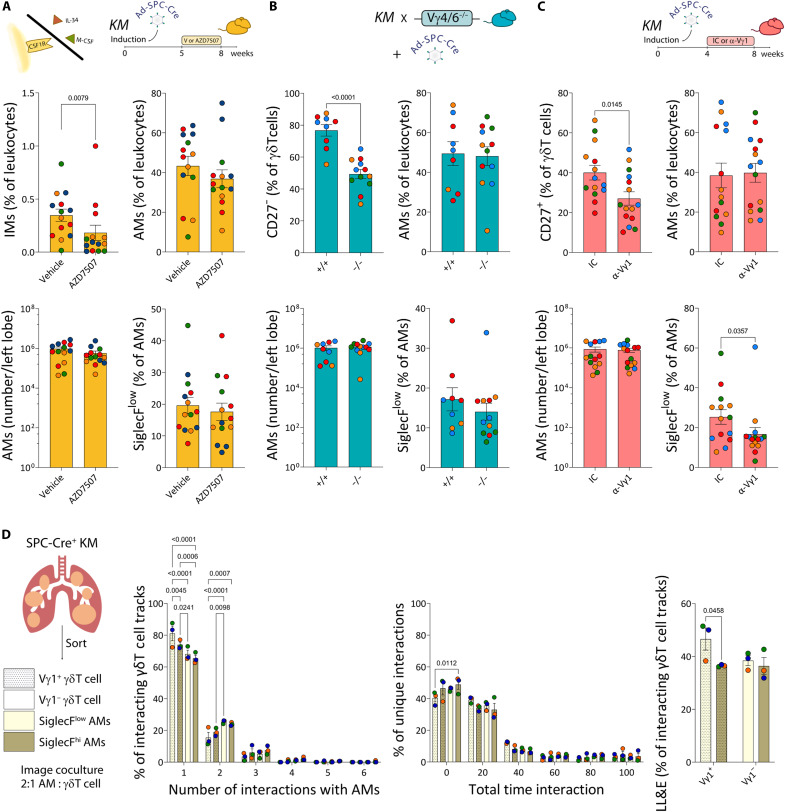
CSF1R-independent SiglecF^low^ AM accumulation is driven by Vγ1 γδ T cells. (**A**) SPC-Cre^+^ KM mice were treated with vehicle or AZD7507 for 3 weeks before analysis of lungs at week 8 postinduction (*n* = 14 mice per group). IMs (% of leukocytes). AMs (% of leukocytes), total number/left lobe, and SiglecF^low^ cells (% of AMs), flow cytometry. M-CSF, macrophage colony-stimulating factor. (**B**) Lungs from KM;*Vγ4/6*^+/+^ and KM;*Vγ4/6*^−/−^ mice 8 weeks postinduction (*n* = 9 to 12 mice per group). CD27^−^ cells (% of γδ Τ cells). AMs (% of leukocytes), total number/left lobe, and SiglecF^low^ cells (% of AMs), flow cytometry. (**C**) SPC-Cre^+^ KM mice were treated with isotype control (IC) or α-Vγ1 mAb for 4 weeks before analysis of lungs at week 8 postinduction (*n* = 14 to 15 mice per group). CD27^+^ cells (% of γδ Τ cells). AMs (% of leukocytes), total number/left lobe, and SiglecF^low^ cells (% of AMs), flow cytometry. (**D**) Vγ1^+^ and Vγ1^−^ γδ T cells, along with SiglecF^low^ and SiglecF^hi^ AMs were sorted from lungs of SPC-Cre^+^ KM mice and imaged in coculture for 100 min (*n* = 3). Percentage of γδ T cells interacting with different numbers of AMs (from 1 to 6) of the total γδ T cells that had any interaction. Percentage of exclusive interactions between γδ T cells and AMs lasting different durations (0 to 100 min, in 20-min bins) of the total number of exclusive pairs. Percentage of LL&E interactions of the total γδ T cells that had any interaction. Each dot represents a mouse, colored by experiment. Data are presented as means ± SEM. Data were analyzed by *t* test if Gaussian distribution (B and C, top), Mann-Whitney test otherwise (A and C, bottom), two-way ANOVA followed by Tukey’s multiple comparisons posttest (D, left and middle) or Fisher’s LSD multiple comparisons posttest (D, right).

As a CSF1-producing γδ Τ cell subset was recently described in a model of malaria (*36*), and tumors can use similar mechanisms to parasitic diseases (*37*–*39*), we next explored whether this molecule was involved in the accumulation of SiglecF^low^ AMs. Thus, we transiently blocked CSF1R in γδ Τ cell–sufficient tumor-bearing mice and evaluated the AM phenotype as before. Consistent with their reliance on CSF1R, we found that administration of the CSF1R inhibitor AZD7507 led to a reduction in IMs. However, we found no changes among SiglecF-defined AMs (Fig. 5A).

Beyond the more functional segregation of γδ Τ cells based on CD27 expression, γδ Τ cells can be further divided according to their γ chain usage. Given that the γδ-TCR clustered during AM-γδ T cell interactions, we next asked whether there was a particular subset of γδ Τ cells responsible for the accumulation of SiglecF^low^ AMs. To this end, we targeted different Vγ chains either crossing KM mice with *Vγ4/6*^−/−^ mice or administering anti-Vγ1 antibody (clone 2.11). In tumor-bearing mice induced as before, we observed an almost twofold reduction in CD27^−^ γδ Τ cells, but AMs and SiglecF^low^ AMs remained the same between *Vγ4/6*^+/+^ and *Vγ4/6*^−/−^ tumor-bearing mice (Fig. 5B). Therefore, these data dismissed a role for Vγ4^+^ and Vγ6^+^ cells in the regulation of SiglecF^low^ AMs. Transient depletion of Vγ1 T cells in tumor-bearing mice reduced the proportion of CD27^+^ γδ Τ cells (Fig. 5C). Although there were no changes in the proportion or total number of AMs between Vγ1^+^ cell-depleted and isotype control–treated tumor-bearing mice, the mice receiving depleting antibodies displayed a decreased percentage of SiglecF^low^ AMs compared to isotype control–treated mice (Fig. 5C). Thus, these data indicate that Vγ1^+^ cells promote the accumulation of SiglecF^low^ AMs in lung tumor-bearing mice in a CSF1R-independent manner.

Last, to explore the interaction dynamics between γδ Τ cells and AMs ex vivo, we FACS sorted Vγ1^+^ and Vγ1^−^ γδ Τ cells and SiglecF^low^ and SiglecF^hi^ AMs from tumor-bearing lungs to image cocultures of the different combinations. Segmentation and tracking of the cells allowed us to detect and quantify the interactions between hundreds of individual γδ Τ cell–AM pairs (Table 2). Among the interacting γδ Τ cells, more Vγ1^+^ γδ Τ cells interacted exclusively with an individual SiglecF^low^ AM. This was in contrast with the three other combinations that interacted preferentially with more than one AM. Vγ1 γδ Τ cell and SiglecF^low^ AM interactions also tended to last longer. When we combined these parameters and quantified “long-lasting and exclusive (LL&E)” interactions, these were significantly more common for the Vγ1^+^ γδ Τ cell^−^SiglecF^low^ AM pairs (Fig. 5D and movie S5). Together, our image analysis pipeline uncovered that Vγ1^+^ γδ Τ cells and SiglecF^low^ AM have a propensity to interact on a cell intrinsic level that does not absolutely require the continued presence of other microenvironmental factors.

**Table 2.** Total number of tracks per experiment.

	SiglecF^low^ AM—Vg1^+^			SiglecF^hi^ AM—Vg1^+^			SiglecF^low^ AM—Vg1^−^			SiglecF^hi^ AM—Vg1^−^		
	Exp. 1	Exp. 2	Exp. 3	Exp. 1	Exp. 2	Exp. 3	Exp. 1	Exp. 2	Exp. 3	Exp. 1	Exp. 2	Exp. 3
AM tracks	522	359	417	537	357	506	582	427	452	519	471	497
γδ T cell tracks	283	331	236	280	291	185	332	334	296	309	247	233
γδ T cell tracks interacting with AMs	141	105	84	118	144	103	184	170	168	179	143	135

## DISCUSSION

**Fig. 6.**
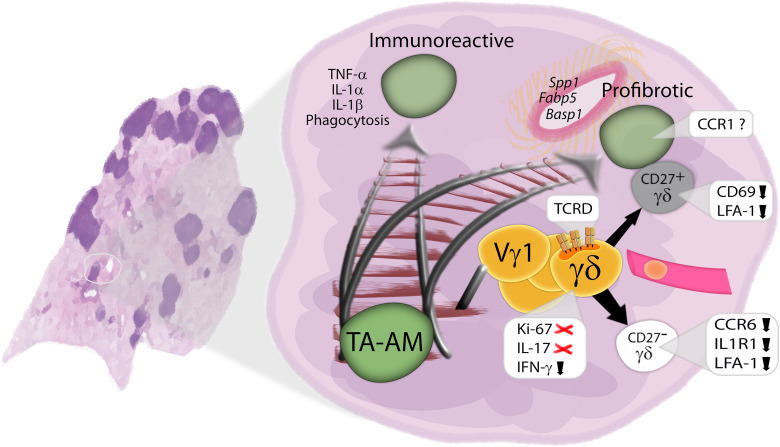
Proposed model of Vγ1 γδ T cells shaping tumor-associated AM function. In the absence of γδ T cells, tumor-associated (TA) AMs produce more inflammatory cytokines and are more phagocytic. Upon recruitment to the TME, γδ T cells contact TA-AM, where TCRD clustering is observed. Vγ1 γδ T cells promote the accumulation of profibrotic AMs expressing *Spp1*, *Fabp5*, and *Basp1*.

AMs are key players in lung repair but can also drive pathological remodeling in acute and chronic inflammation. In particular, the accumulation of SiglecF^low^ AMs has been implicated in lung fibrosis (*11*). Here, we demonstrated that Vγ1 γδ T cells promote the accumulation of these cells, which we speculate are of monocyte origin. Because of our autochthonous LuAd model’s reliance on Cre delivery, we were unable to confirm this hypothesis using Cre-based fate mapping models. However, extensive evidence suggests that macrophages with a CD11c^hi^ phenotype and lower levels of SiglecF are reflective of monocyte-derived cells in a transitional state (*11*, *12*, *30*, *40*). This has been corroborated by various fate mapping approaches, including tissue-protected bone marrow chimeras, busulfan-conditioned chimeras, and genetic lineage tracing (e.g., with *Ms4a3*-Cre mice). Mo-AMs have been shown to exhibit either immunoreactive or profibrotic traits, depending on the insult (*13*). In lung cancer, the factors that control these divergent functional fates remain unidentified. Our findings implicate γδ T cells as prime suspects: In the absence of γδ T cells, the ECM has a different texture, AMs produce more inflammatory cytokines in response to LPS, and they display more phagocytic activity against bacteria. At the molecular level, as we observed TCRD clustering consistent with immunological synapse formation, we propose that γδ-TCR engagement, in particular the complex expressing the Vγ1 chain, may contribute to steering the functional programs of TA-AMs (Fig. 6).

Thus, the work presented here broadens our understanding of key interactions that shape the immune microenvironment in lung cancer, beyond a focus on murine tumor growth per se. These insights may have implications for patient morbidity and could influence therapeutic efficacy and susceptibility to lung infections, ultimately affecting patient outcomes. This is very timely as there is substantial interest in exploiting γδ T cells for cancer therapy (*41*).

An open question is whether the increased γδ T cells displaying phenotypic changes represent the recruitment of a distinct subset or instead adapt their phenotype upon entry into the TME. Although we cannot fully exclude heterogeneity, γδ T cells in peripheral blood were indistinguishable between tumor-bearing and control mice, arguing against the preferential recruitment of a preexisting circulating subset. In this context, we think our CellChat analysis using steady-state γδ T cells provides a reasonable approximation of newly recruited cells while acknowledging that tissue-derived cues likely imprint their phenotype.

Another consideration relates to our use of full-body *Tcrd* knockout mice, where observed effects could reflect compensatory mechanisms instead of a genuine contribution of γδ T cells (*42*, *43*). However, the absence of an effect in full-body *Vγ4/6* knockout mice, together with the recapitulation of a decrease in SiglecF^low^ AMs following transient anti-Vγ1 antibody treatment, strengthens the interpretation that these effects are direct rather than indirect. It is worth noting that, although clone 2.11 has been widely used for depletion (*44*, *45*), other monoclonal antibodies (mAbs) against the γδ-TCR have been shown to induce internalization of the receptor rather than cell depletion (*46*). If the antibody internalizes the γδ-TCR complex expressing the Vγ1 chain without depleting the cell yet still affects SiglecF^low^ AMs, this would provide functional validation of the γδ-TCR role, complementing our demonstration of its clustering during AM-γδ T cell interactions.

In a different GEMM of LuAd driven by the mutation *Kras^G12D^* and *Tp53* loss, γδ T cells recapitulated the classic circuit of IL-17 driven protumor neutrophils (*9*). However, in our model driven by *Kras^G12D^* and *MYC*, we have not observed an expansion in the proportion of γδ T cells producing IL-17 nor an increase in neutrophils at the time points studied. Similarly, no analogous population of IL-17–producing γδ T cells have been reported in human lung cancer. A subset of Vδ1 γδ T cells with wound healing functions that correlates with poor prognosis in human colorectal cancer has been recently identified (*47*). It would be important to determine whether a similar subset exists in lung cancer and whether it might rely on tumor MYC, a commonly dysregulated gene in cancer that modulates the immune microenvironment (*48*–*51*). Given also that never-smokers often have unmutated p53 (*52*), this underscores the need to account for a variety of mutational makeups in preclinical models. Doing so can help ensure that the literature better reflects the disease’s heterogeneity.

In summary, we report that, in our GEMM of LuAd, γδ Τ cells are recruited to tumors, where the CD27^+^ subset interacts preferentially with TAMs. We speculate that this interplay imprints AM function as we demonstrated that Vγ1 cells mediate the accumulation of SiglecF^low^ AMs, which feature a profibrotic profile. Moreover, we identify γδ-TCR clustering during AM-γδ T cell interactions, suggesting a potential mechanism by which γδ T cells shape AM responses (Fig. 6). These findings could be relevant to better understand radiation-induced pulmonary fibrosis (*53*), paraneoplastic syndromes, and common comorbidities such as pneumonia that often cost the lives of patients with cancer (*54*). Determining whether, and which, human γδ T cells shape TAMs analogously to our mouse model may help to refine immunotherapeutic approaches in LuAd.

## MATERIALS AND METHODS

### Experimental design

The main aim of this study was to characterize and locate γδ Τ cells and their interactions in an autochthonous model of LuAd driven by *Kras^G12D^* and *MYC*. To this end, we used flow cytometry, confocal imaging, and scRNA-seq data. Identification of interactions between γδ Τ cells and macrophages prompted scRNA-seq analysis of macrophages. We performed further transcriptomic and functional analysis of AMs in the context of γδ Τ cell deficiency to assess how they are influenced by γδ Τ cells.

### Mouse models

The *LSL*–*Kras^G12D^* (*55*), *Rosa26*^*DM.lsl*–*MYC*^ (Kruspig *et al.*, 2018; RRID: IMSR_JAX:033805), *Tcrd^tm1Mom^* (*56*), and *Tcrg^tm1Iku^* referred as *Vγ4/6* (*57*) alleles were previously described. All genetically modified mice were bred in-house, maintained on a mixed FVB/N, C57BL/6 background, on a 12-hour/12-hour light/dark cycle, and fed/watered ad libitum under specific pathogen–free conditions. Both males and females were included in approximately equal numbers, and mice were randomly assigned to induction or treatment cohorts, balanced only for sex. KM mice were crossed with *iCcr*-*rep*^+/+^ mice (gifted to us by G. J. Graham, School of Infection and Immunity, University of Glasgow, Glasgow, UK), with *Tcrd*^−/−^ mice (gifted to us by A. Hayday, The Francis Crick Institute, London, UK), or with *Vγ4/6*^−/−^ mice (gifted to us by R. O’Brien, National Jewish Health, Denver, Colorado, USA). Control FVB/N mice were purchased from Charles River. All genotyping was performed by TransnetYX Inc.

Recombinant adenovirus Ad5mSPC-Cre, purchased from The University of Iowa gene therapy facility, was used to initiate LuAd by removing *lsl* cassettes from conditional alleles in alveolar type 2 *Spc* expressing cells. A total of 1 × 10^8^ PFU (plaque-forming units) of Ad5mSPC-Cre viral particles were administered intranasally in 4 mM CaCl_2_ (Sigma-Aldrich) minimum essential medium (MEM) (Thermo Fisher Scientific) to young adult (10- to 12-week-old) mice sedated with medetomidine and ketamine (*58*). Atipamezole was administered to reverse anesthesia.

For depletion of Vγ1^+^ cells, mice were injected intraperitoneally with anti-Vγ1 antibody (clone 2.11, BioXcell) beginning 4 weeks after allele activation with a single dose of 200 μg, followed by 100 μg twice per week thereafter. Control mice followed the same dosage regime with isotype control (polyclonal Armenian hamster IgG, BioXcell).

The CSF1R inhibitor (AZD7507) was provided by AstraZeneca under a research collaboration agreement with the CRUK Scotland Institute. Beginning 5 weeks after allele activation, AZD7507, dissolved in 0.5% hydroxypropyl methylcellulose (HPMC) and 0.1% Tween 80 in distilled water, was administered at 100 mg/kg by oral gavage twice daily for 1 week and once per day thereafter. Control mice followed the same dosage regime with vehicle.

All mice were humanely euthanized by overdose of pentobarbital 8 weeks after allele activation, and tissues (spleen, blood, and lungs) were harvested for downstream analyses. Procedures were performed in accordance with UK Home Office license numbers 70/7950 and PE47BC0BF at the CRUK Scotland Institute and approved by the University of Glasgow animal Welfare and Ethical Review Board (AWERB).

### Tissue processing

Blood samples were withdrawn from the femoral artery using capillary action collection tubes with EDTA, and red blood cells were lysed using ammonium-chloride-potassium buffer in 96-well V-bottom plates. Spleens were mashed through a 70-μm filter with 5 ml of Dulbecco’s modified Eagle’s medium (DMEM) with 5% fetal bovine serum (FBS) and 1% penicillin/streptomycin (Life Technologies). Before excising lungs, a small incision was made in the trachea, and a customized blunted needle was inserted. For some experiments, the left bronchus was tied with a suture and, subsequently, 0.6 ml of 2% low-melting-point agarose in phosphate-buffered saline (PBS) was instilled slowly through the needle (1.1 ml in cases where whole lungs were inflated). Agarose-inflated lungs were processed for confocal microscopy. Agarose-free left lobe or whole lungs were minced with scissors and incubated at 37°C with agitation in RPMI with 10% FBS, before mashing through a 70-μm filter. For scRNA-seq experiments, lungs were microdissected under a stereomicroscope. For the SPC-Cre^+^ KM tumor total cells dataset, enzymatic dissociation with collagenase D (1 μg/ml; Roche) and DNAse I (25 μg/ml; Thermo Fisher Scientific) was carried out using the gentleMACS Octo Dissociator (Miltenyi Biotec). Then, tumor suspensions were mashed through a 70-μm filter and live cells were enriched using debris removal solution (Miltenyi Biotec). For obtaining single-cell suspensions for the KM macrophage datasets, microdissected tissue was processed in the same way as minced lungs. Before sorting, single-cell suspensions were layered onto a Percoll (GE Healthcare) gradient 40/80 to eliminate debris and epithelial cells. For histology, whole lungs were inflated through the trachea with 10% neutral buffered formalin and fixed for 24 hours, before transferring to 70% ethanol.

### Flow cytometry

For intracellular staining, single-cell suspensions from lungs were incubated with Cell Stimulation Cocktail (eBioscience) in 96-well V-bottom plates at 37°C for 3 hours. In one of the three experiments, a Percoll (GE Healthcare) gradient step was included before plating. After stimulation, cells were stained with a viability dye for 15 min at room temperature, followed by blocking with TruStain FCX PLUS (BioLegend), and then staining of surface markers in Brilliant stain buffer (BD Biosciences). After washing, cells were fixed and permeabilized in FOXP3 Transcription Factor Fixation/Permeabilization solution (Thermo Fisher Scientific) following the manufacturer’s instructions. Antibodies for intracellular antigens were prepared in permeabilization buffer, and cells were incubated for 30 min at 4°C. When assessing only surface markers, cells were similarly blocked, stained for viability, and then stained with a combination of antibodies detailed in Table 1. For transcriptomic and functional experiments, cells were sorted using a FACSAria II cell sorter (BD). For phenotyping, cells were fixed in 2% formaldehyde (VWR) and analyzed using an LSR Fortessa analyzer (BD) within a week. Just before acquisition, SPHERO ACCUCOUNT fluorescent beads (Spherotech) were added for quantification purposes. Data were analyzed using FlowJo software (BD, version 10.8.1).

### PCLS and confocal imaging

Fresh agarose-inflated lungs were sliced into 300-μm-thick sections on a vibrating microtome (Campden Ltd.). For γδ Τ cell mapping, slices were blocked with TruStain FCX PLUS (BioLegend) and stained with anti TCRD AF647 (GL3, BioLegend), CD27 AF594 (LG.3A10, BioLegend), CD31 AF488 (390, BioLegend), and CD45 AF700 (30-F11, BioLegend) antibodies in complete medium (phenol red–free DMEM supplemented with 1% FBS, Gibco) for 20 min at 37°C. After washing, slices were fixed in 4% formaldehyde in PBS (VWR) for 20 min at room temperature. Then, they were permeabilized and blocked in PBS with 0.3% Triton X-100 (Sigma-Aldrich), 10% normal goat serum (Sigma-Aldrich), 1% bovine serum albumin (BSA) (Sigma-Aldrich), and 0.001% sodium azide (VWR, UK) and subsequently stained with CD68 BV421 (FA-11, BioLegend) overnight at 4°C. Last, slices were mounted in Ce3D tissue clearing solution (BioLegend) and imaged on an LSM 880 NLO upright confocal/multiphoton microscope (Carl Zeiss) equipped with a 32-channel gallium arsenide phosphide (GaAsP) spectral detector using 20×/1–numerical aperture (NA) water immersion objective lens. Positions were selected based only on the topological features and excited simultaneously with 405, 488, 561, and 633 continuous wave laser lines and then with a pulsed two-photon laser tuned to 950 nm for second harmonic generation imaging of fibrillar collagen. For both acquisitions, signal was collected onto the 32 GaAsP detector linear array in lambda mode with a resolution of 8.9 nm over the visible spectrum. Spectral images were added together after linear unmixing with Zen software (Carl Zeiss) using reference spectra acquired from unstained tissues for autofluorescence and beads labeled with conjugated antibodies for each fluorophore. For CCR1^+^ macrophage mapping, the same protocol was applied to formaldehyde-fixed lungs. Slices were stained with anti-CD68 AF700 (FA-11, BioLegend) and SYTOX Blue Nucleic Acid Stain (Invitrogen). The presence of mRuby2, mTagBFP2, iRFP682, and Clover in the reporter mice precluded reliable CD31 unmixing, so it was excluded from analysis. Alternatively, for live imaging, slices were stained with anti-CD31 AF647 or AF488 (clone 390, BioLegend), TCRD AF488 or AF647 (GL3, BioLegend), and CD11c AF594 (N418, BioLegend) antibodies and Hoechst 33342 (Thermo Fisher Scientific, UK) in complete medium for 20 min at 37°C. Slices were imaged on an LSM880 inverted confocal microscope with Airyscan detectors (Carl Zeiss) in a full incubation chamber at 37°C with 5% CO_2_. Airyscan time-lapse sequence processing was performed in Zen software (Carl Zeiss). Data were analyzed with Imaris software (Bitplane, Oxford Instruments, version 10.0.0).

### scRNA sequencing

Cells were resuspended in PBS containing 0.05% BSA and counted using a hemocytometer. A total of 40,000 cells were loaded onto each channel of Chromium Chip G using reagents from the 10x Chromium Single-Cell 3′ v3 Bead Kit and Library (10X Genomics) according to the manufacturer’s protocol. The libraries were analyzed using the Bioanalyzer High Sensitivity DNA Kit (Agilent Technologies). scRNA-seq libraries were sequenced on the Illumina NovaSeq 6000 with paired-end 150–base pair (bp) reads. Quality checks and trimming of the raw scRNA-seq fastq data files were done using FastQC (*59*), FastP (*60*), and FastQ Screen (*61*, *62*). The creation of the transgenic reference genome and annotation was based on the GRCm38 version of the mouse genome and annotation (*63*). Alignment to the reference genome and aggregation were completed using 10x Genomics Cell Ranger version 3.1.0. Quality control, integration of data, clustering, marker gene identification, and exploratory analysis was accomplished using the Seurat package version 4 (*25*) and the R environment version 4. Identification of high-quality cells used the adaptive thresholds method as outlined in (*64*).

For the Seurat object of SPC-Cre^+^ KM tumors, 20 principal components were retained for clustering at 0.1 resolution. For the Seurat object of pulmonary mouse γδ Τ cell (ArrayExpress, #E-MTAB-10677), 15 principal components were retained for clustering at a 0.2 resolution. Both datasets were appended with the Seurat command “merge” and then normalized and scaled (regressing out RNA counts) for subsequent analysis using CellChat (*24*).

The Seurat objects of KM macrophages and pulmonary myeloid cells (GSE181894) were integrated following normalization with SCTransform. Clustering was performed with 30 principal components at a 0.1 resolution. Cluster 0 (AM) was subset and used to build a heatmap with the 20 top positive markers of “Condition.” The signature corresponding to “Profibrotic AM” was assessed using “AddModuleScore.” We reclustered SPC-Cre^+^ AMs, taking 30 principal components at a 0.15 resolution. For slingshot pipeline, cluster 0.0 was set as start. Differentially up-regulated genes were obtained using “FindAllMarkers” with the test MAST. These genes were converted to human orthologous symbols using the library nichenetr (*65*) to interrogate the GEPIA2 database (*32*). The R package Escape version 3.17 was used to calculate ssGSEA (*66*) using gene sets from the Molecular Signatures Database (*67*). The Mann-Whitney *U* test was used to determine significant differences. The ReactomeGSA package (*68*) was used to perform GSVA (*69*) using data from the Reactome pathway knowledgebase (*70*).

All computational analysis was documented at each stage using MultiQC (version 1.8) (*71*), Jupyter Notebooks (*72*), and/or R Notebooks (*73*).

### Bulk RNA-seq

Pellets of 150,000 to 250,000 cells were resuspended in buffer RLT (Qiagen) and stored at −70°C until RNA was isolated using the RNeasy Micro Kit (Qiagen) following the manufacturer’s instructions. Quality control of all RNA samples was performed (Agilent TapeStation 4200, High Sensitivity RNA ScreenTape), and samples showed RNA Integrity Number (RIN) values > 8. RNA concentrations were determined by Qubit Fluorometer using the Qubit RNA Broad Range assay (both Thermo Fisher Scientific), with 150 ng of total RNA used as initial input. Libraries were then prepared using the manufacturer’s standard procedures (Illumina Stranded mRNA), with IDT for Illumina RNA UD Indexes used to index libraries. Post library QC was then performed using High Sensitivity D1000 ScreenTape (Agilent) for library sizing and profiling and quantified using the Qubit High Sensitivity DNA assay. Libraries were then pooled equimolar, to a final concentration of 4 nM before sequencing. The library pool was then sequenced on an Illumina NextSeq 500 instrument, on a High-Output 150 cycle run with a paired-end 74-bp read length.

Quality checks and trimming on the raw RNA-seq data files were done using FastQC version 0.12.1 (*59*), FastP version 0.23.4 (*60*), and FastQ Screen version 0.15.1 (*61*). RNA-seq paired-end reads were aligned to the GRCm39.110 version of the mouse genome and annotation (*74*), using STAR version 2.7.11b (*75*). Aligned genes were identified using Feature Counts from the SubRead package version 2.0.6 (*76*). Expression levels were determined and statistically analyzed using the R environment version 4.3.2 (*77*) and using packages from the Bioconductor data analysis suite (*78*). Differential gene expression was analyzed on the basis of the negative binomial distribution using the DESeq2 package version 1.42.0 (*79*) and adaptive shrinkage using Apelgm (*80*). Significant genes from the DEG analysis were those identified as having an absolute fold change above 1.5 and adjusted *P* value equal to or below 0.05.

Then, DEGs were assessed using “AddModuleScore” function in the Seurat package into the scRNA-seq clusters. The Mann-Whitney *U* test was used to determine whether the module scores were significantly different between clusters. Identification of enriched biological functions was achieved using g:Profiler (*81*), GESA version 7.2 from the Broad Institute (*67*), and the R package rrvgo (*82*).

Computational analysis was documented at each stage using MultiQC (version 1.8) (*71*), Jupyter Notebooks (*72*), and R Notebooks (*73*).

### Histology

Lungs were loaded into labeled histology processing cassettes and processed through a series of graded ethanol (70, 90, 95, and 100%, x3), three changes of xylene, and three changes of histology wax applied under pressure. This was performed using an overnight tissue processing cycle on an Epredia Excelsior tissue processor. Following this, the tissue was orientated and embedded in histology wax to create a formalin-fixed paraffin-embedded (FFPE) block. Four-micrometer sections were cut from each FFPE block and placed onto a glass slide, and the sections were oven-dried at 60°C for 2 hours.

Hematoxylin and eosin (H&E) staining was performed on a Leica autostainer (ST5020). Sections were dewaxed in xylene, taken through graded ethanol solutions and stained with Haem Z (RBA-4201-00A, CellPath) for 13 min. Sections were washed in water, differentiated in 1% acid alcohol, washed, and nuclei blued in Scott’s tap water substitute (in-house). After washing with tap water, sections were placed in Putt’s Eosin (in-house) for 3 min. The H&E sections were washed in tap water, dehydrated through graded ethanol solutions, and placed in xylene. Staining for Sirius Red was performed manually on FFPE sections that were dewaxed in xylene and taken through graded alcohols before washing in water. Rehydrated slides were stained for 2 hours in Sirius Red staining solution. The Sirius Red staining solution was equal volumes of 0.1% Direct red 80 (365548, Sigma-Aldrich) and 0.1% Fast green (S142.2, Raymond A. Lamb) (both in distilled water) combined in a 1:9 dilution with aqueous picric acid solution (84512.260, VWR). The stained sections were coverslipped from xylene using the DPX mountant (SEA-1300-00A, CellPath). Once the DPX mountant had solidified, the glass slides were loaded onto a Leica Aperio AT2 slide scanner, and digital images captured at x20 magnification.

Tumor burden was determined for each mouse using QuPath version 0.3.2 (*83*) as the % of lung tissue occupied by tumors, averaging H&E slides from three paraffin-embedded sections at 400-μm intervals.

The analysis of structural characteristics of collagen fibers was adapted from our previous work (*35*) to be used on lung sections stained with Sirus Red. Collagen-specific staining was deconvolved from bright-field images using QuPath (*83*). For each slide, five regions of tumor and parenchyma (400 μm by 260 μm) were exported in Fiji. GLCM texture analysis derives statistical measures by considering the spatial relationship of pixels in an image, so-called “second-order statistics.” The features measured using the GLCM plug-in included contrast, entropy, correlation, inverse difference moment, and angular second moment. Per mouse, the values for the five regions were averaged and then normalized to the average of wild-type parenchyma in each independent experiment to mitigate batch effects due to background differences.

### Functional assays

Sorted AMs from SPC-Cre^+^ KM; *Tcrd* knockout and wild-type mice were stimulated in vitro with either LPS (100 ng/ml, from *E. coli* O111:B4, Sigma-Aldrich), 10,000 KM cells (a cell line derived from an SPC-Cre^+^ KM LuAd), or control medium. A total of 40,000 AMs were plated in duplicate in 96-well plates for each condition. Cell-free supernatants were harvested 24 hours later and stored at −70°C until cytokines were assessed using the LEGENDplex mouse inflammation panel (BioLegend), following the manufacturer’s instructions. Samples were run in an Attune NxT flow cytometer (Thermo Fisher Scientific), and data were analyzed with the LEGENDplex Data Analysis Software Suite (cloud version, release 15 May 2024).

For the phagocytosis assay, a total of 40,000 AMs from each genotype were plated in duplicate in 96-well glass-bottom plates coated with poly-l-lysine solution (Sigma-Aldrich). After a 3-hour incubation with Red *E. coli* BioParticles (Thermo Fisher Scientific) or control medium, cells were fixed in 4% formaldehyde (VWR) and stained with 4′,6-diamidino-2-phenylindole (DAPI) and HCS CellMask Deep Red (Thermo Fisher Scientific). Cells were imaged on an Opera Phenix high-content imaging microscope (Revvity). For quantitation, 25 fields at 20x were imaged per well (lowest total sample size per condition = 11,869 cells). Cells were segmented using Harmony 4.9 image analysis software (Revvity). The pixel sum of intensity (Ex561nm) per cytoplasm, which excludes the nucleus, was calculated for each cell and a mean value of all cells calculated from this. To confirm that BioParticles were inside the cytoplasm, representative images were taken at 63x magnification with a *z*-stack covering 10.5 μm.

### TCRD clustering analysis

Ten-minute time-lapse imaging sequences were acquired from live PCLSs of three different lung lobes per mouse as described before. Quality control and classification of time-lapse sequences as “No AM contact” or “AM contact” were performed using Imaris Software (Bitplane, Oxford Instruments, version 10.0.0). For each time point, maximum intensity projections (MIPs) preserving the original intensity values were generated for the TCRD channel.

Fiji (version 1.54p 17) was used to define the regions of interest (ROIs) across all time points and all MIP images (*84*). These ROIs were generated using an ImageJ macro that ensured consistent size and shape across all images. ROIs were manually placed to cover the cell and a representative background area and then exported as ImageJ ROIs for blinded analysis using an automated Python (version 3.10) pipeline in a Jupyter Notebook. Briefly, a mask was generated for the pixels within the cell ROIs above a threshold of five times the mean background intensity. To improve the quality of the segmentation mask, a filter was applied to remove all masks smaller than 10 px to ensure only meaningful regions within the cell were analyzed. Local maxima within a 5-px radius were defined as peaks using the “peak_local_max” function within the scikit-image package (*85*). Properties of the masked area for all images and time points were exported to an Excel file for further analysis, along with the cropped images of the cell and the background, and the masks used to define the area of the cell analyzed. The scripts used for this analysis can be found on GitHub in the following repository: https://github.com/Beatson-CompBio/Raffo-Iraolagoitia_2024_A.

### Interaction analysis

Sorted SiglecF^low^ and SiglecF^hi^ AMs from SPC-Cre^+^ KM mice were stained with CFSE (carboxyfluorescein diacetate succinimidyl ester; Thermo Fisher Scientific) and cocultured with either sorted Vγ1 γδ T cells or not-Vγ1 γδ T cells from the same mouse at a 2:1 ratio (5000 AMs with 2500 γδ T cells) in 384-well glass-bottom plates in the presence of Cytotox Red dye (Sartorius). Phase, green (441 to 481|503 to 544 nm), and red (567 to 607|622 to 704 nm) channel images were taken every 2 min for a total of 100 min using a IncuCyte S3 system (Sartorius) with a ×10 objective.

A GitHub repository can be found at the following location: https://github.com/Beatson-CompBio/Raffo-Iraolagoitia_2024_A, which contains the scripts used to analyze the interaction between AMs and γδ T cells. Drift correction was performed with the Fiji plug-in “Correct 3D drift” (*86*). Cells were segmented in the phase-contrast images using a bespoke deep learning model developed by retraining the “livecell” model, available as part of the cellpose model zoo (*87*). The average precision of the retrained model was 0.94 when tested on unseen data. The green and red channels were segmented to identify the AMs and dead cells, respectively. If cells were not AMs nor dead, then they were designated as “γδ T cell.” An additional size filter was implemented. Then, cell tracks were calculated using the Python package btrack (*88*). For each frame in the time-lapse experiment, the masks for the three different cell types were analyzed to see whether the γδ T cells encounter AMs or dead cells across the experiment and recorded how long the interactions lasted and how many each cell experiences for each of the cell types.

### Use of ChatGPT

ChatGPT-4 was used to help to edit text for brevity during the drafting process using prompts like: [can you say “two-way analysis of variance (ANOVA) with Sidak’s multiple comparisons posttest “with less words]. It was also used to edit sections of the draft manuscript where key information (e.g., cell type or marker) was replaced with “A,” “B,” etc. Prompts such as: [Is this a good first paragraph for a discussion section on a paper: “....] were used to check that the key messages in the paper were clear and being comprehended by the model; if not, the text in the manuscript was further refined manually. Suggestions on punctuation and synonyms were sometimes used directly from this process. Once drafted, all authors further edited for clarity and the manuscript was examined using iThenticate to avoid any inadvertent plagiarism.

ChatGPT-4 was also used in the initial stages of developing the ImageJ macro used in the “TCRD clustering analysis.” The prompt [can I create a ROI of fixed area?] was used to form the start of a macro that was then refined and implemented. The final macro “Create fixed-size ROIs for each time point with correct naming.ijm” is given in full at the Zenodo location associated with this paper (DOI: 10.5281/zenodo.17077114).

### Statistical analysis

Normalized total number of cells was calculated as the total number of cells in a sample, divided by the average total number of cells in control samples for each independent experiment; this allowed us to combine two experiments where whole lungs were processed for flow cytometry with one experiment where the right lobes were used for imaging. Mean fluorescence intensity (MFI) relative change was calculated as the difference between MFI of the gated population and the average MFI of the same gating in control samples, divided by the average MFI of the same gating in control samples, for each independent experiment. Statistical analysis was performed using GraphPad Prism (version 9.5.1) as detailed in the figure legends.

## References

[1] C. M. Lloyd, B. J. Marsland, Review lung homeostasis: Influence of age, microbes, and the immune system. Immunity 46, 549–561 (2017). 28423336 10.1016/j.immuni.2017.04.005

[2] J. G. Borger, Spatiotemporal cellular networks maintain immune homeostasis in the lung. EMJ Respir. 8, 108–119 (2020).

[3] M. W. Dahlgren, A. B. Molofsky, Adventitial cuffs: Regional hubs for tissue immunity. Trends Immunol. 40, 877–887 (2019). 31522963 10.1016/j.it.2019.08.002 PMC6823140

[4] S. C. Edwards, A. Hedley, W. H. Hoevenaar, R. Wiesheu, T. Glauner, A. Kilbey, R. Shaw, K. Boufea, N. Batada, S. Hatano, Y. Yoshikai, K. Blyth, C. Miller, K. Kirschner, S. B. Coffelt, PD-1 and TIM-3 differentially regulate subsets of mouse IL-17A-producing γδ T cells. J. Exp. Med. 220, e20211431 (2023). 36480166 10.1084/jem.20211431 PMC9732671

[5] J. C. Ribot, N. Lopes, B. Silva-Santos, γδ T cells in tissue physiology and surveillance. Nat. Rev. Immunol. 21, 221–232 (2021). 33057185 10.1038/s41577-020-00452-4

[6] Y. Wu, D. Biswas, I. Usaite, M. Angelova, S. Boeing, T. Karasaki, S. Veeriah, J. Czyzewska-Khan, C. Morton, M. Joseph, S. Hessey, J. Reading, A. Georgiou, M. Al-Bakir, TRACERx Consortium, N. McGranahan, M. Jamal-Hanjani, S. A. Quezada, A. C. Hayday, C. Swanton, A local human Vδ1 T cell population is associated with survival in nonsmall-cell lung cancer. Nat. Cancer 3, 696–709 (2022). 35637401 10.1038/s43018-022-00376-z PMC9236901

[7] B. Silva-Santos, S. Mensurado, S. B. Coffelt, γδ T cells: Pleiotropic immune effectors with therapeutic potential in cancer. Nat. Rev. Cancer 19, 392–404 (2019). 31209264 10.1038/s41568-019-0153-5 PMC7614706

[8] S. B. Coffelt, K. Kersten, C. W. Doornebal, J. Weiden, K. Vrijland, C.-S. Hau, N. J. M. Verstegen, M. Ciampricotti, L. J. A. C. Hawinkels, J. Jonkers, K. E. de Visser, IL-17-producing cd T cells and neutrophils conspire to promote breast cancer metastasis. Nature 522, 345–348 (2015). 25822788 10.1038/nature14282 PMC4475637

[9] C. Jin, G. K. Lagoudas, C. Zhao, P. C. Blainey, J. G. Fox, T. Jacks, Commensal microbiota promote lung cancer development via γδ T cells. Cell 176, 998–1013 (2019). 30712876 10.1016/j.cell.2018.12.040 PMC6691977

[10] J. M. Wands, C. L. Roark, M. K. Aydintug, N. Jin, Y.-S. Hahn, L. Cook, X. Yin, J. Dal Porto, M. Lahn, D. M. Hyde, E. W. Gelfand, R. J. Mason, R. L. O’Brien, W. K. Born, Distribution and leukocyte contacts of γδ T cells in the lung. J. Leukoc. Biol. 78, 1086–1096 (2005). 16204632 10.1189/jlb.0505244

[11] A. V. Misharin, L. Morales-Nebreda, P. A. Reyfman, C. M. Cuda, J. M. Walter, A. C. McQuattie-Pimentel, C.-I. Chen, K. R. Anekalla, N. Joshi, K. J. N. Williams, H. Abdala-Valencia, T. J. Yacoub, M. Chi, S. Chiu, F. J. Gonzalez-Gonzalez, K. Gates, A. P. Lam, T. T. Nicholson, P. J. Homan, S. Soberanes, S. Dominguez, V. K. Morgan, R. Saber, A. Shaffer, M. Hinchcliff, S. A. Marshall, A. Bharat, S. Berdnikovs, S. M. Bhorade, E. T. Bartom, R. I. Morimoto, W. E. Balch, J. I. Sznajder, N. S. Chandel, G. M. Mutlu, M. Jain, C. J. Gottardi, B. D. Singer, K. M. Ridge, N. Bagheri, A. Shilatifard, G. R. Scott Budinger, H. Perlman, Monocyte-derived alveolar macrophages drive lung fibrosis and persist in the lung over the life span. J. Exp. Med. 214, 2387–2404 (2017). 28694385 10.1084/jem.20162152 PMC5551573

[12] H. Aegerter, J. Kulikauskaite, S. Crotta, H. Patel, G. Kelly, E. M. Hessel, M. Mack, S. Beinke, A. Wack, Influenza-induced monocyte-derived alveolar macrophages confer prolonged antibacterial protection. Nat. Immunol. 21, 145–157 (2020). 31932810 10.1038/s41590-019-0568-x PMC6983324

[13] J. Kulikauskaite, A. Wack, Teaching old dogs new tricks? The plasticity of lung alveolar macrophage subsets. Trends Immunol. 41, 864–877 (2020). 32896485 10.1016/j.it.2020.08.008 PMC7472979

[14] M. Casanova-Acebes, E. Dalla, A. M. Leader, J. LeBerichel, J. Nikolic, B. M. Morales, M. Brown, C. Chang, L. Troncoso, S. T. Chen, A. Sastre-Perona, M. D. Park, A. Tabachnikova, M. Dhainaut, P. Hamon, B. Maier, C. M. Sawai, E. Agulló-Pascual, M. Schober, B. D. Brown, B. Reizis, T. Marron, E. Kenigsberg, C. Moussion, P. Benaroch, J. A. Aguirre-Ghiso, M. Merad, Tissue-resident macrophages provide a pro-tumorigenic niche to early NSCLC cells. Nature 595, 578–584 (2021). 34135508 10.1038/s41586-021-03651-8 PMC8923521

[15] M. D. Park, I. Reyes-Torres, J. Leberichel, P. Hamon, N. M. Lamarche, S. Hegde, M. Belabed, L. Troncoso, J. A. Grout, A. Magen, E. Humblin, A. Nair, M. Molgora, J. Hou, J. H. Newman, A. M. Farkas, A. M. Leader, T. Dawson, D. D’souza, S. Hamel, A. Rodriguez Sanchez-Paulete, B. Maier, N. Bhardwaj, J. C. Martin, A. O. Kamphorst, E. Kenigsberg, M. Casanova-Acebes, A. Horowitz, B. D. Brown, L. Ferrari De Andrade, M. Colonna, T. U. Marron, M. Merad, TREM2 macrophages drive NK cell paucity and dysfunction in lung cancer. Nat. Immunol. 24, 792–801 (2023). 37081148 10.1038/s41590-023-01475-4 PMC11088947

[16] G. S. Falchook, M. Peeters, S. Rottey, L. Y. Dirix, R. Obermannova, J. E. Cohen, R. Perets, R. Shapira Frommer, T. M. Bauer, J. S. Wang, R. D. Carvajal, J. Sabari, S. Chapman, W. Zhang, B. Calderon, D. A. Peterson, A phase 1a/1b trial of CSF-1R inhibitor LY3022855 in combination with durvalumab or tremelimumab in patients with advanced solid tumors. Invest. New Drugs 39, 1284–1297 (2021). 33852104 10.1007/s10637-021-01088-4

[17] A. Dowlati, R. Donald Harvey, R. D. Carvajal, O. Hamid, S. J. Klempner, J. S. W. Kauh, D. A. Peterson, D. Yu, S. C. Chapman, A. M. Szpurka, M. Carlsen, T. Quinlan, R. Wesolowski, LY3022855, an anti–colony stimulating factor-1 receptor (CSF-1R) monoclonal antibody, in patients with advanced solid tumors refractory to standard therapy: Phase 1 dose-escalation trial. Invest. New Drugs 39, 1057–1071 (2021). 33624233 10.1007/s10637-021-01084-8

[18] A. R. Razak, J. M. Cleary, V. Moreno, M. Boyer, E. Calvo Aller, W. Edenfield, J. Tie, R. Donald Harvey, A. Rutten, M. A. Shah, A. J. Olszanski, D. Jäger, N. Lakhani, D. P. Ryan, E. Rasmussen, G. Juan, H. Wong, N. Soman, M.-A. Damiette Smit, D. Nagorsen, K. P. Papadopoulos, Safety and efficacy of AMG 820, an anti-colony-stimulating factor 1 receptor antibody, in combination with pembrolizumab in adults with advanced solid tumors. J. Immunother. Cancer 8, 1006 (2020). 10.1136/jitc-2020-001006 PMC7552843 33046621

[19] K. P. Papadopoulos, L. Gluck, L. P. Martin, A. J. Olszanski, A. W. Tolcher, G. Ngarmchamnanrith, E. Rasmussen, B. M. Amore, D. Nagorsen, J. S. Hill, J. Stephenson, Cancer therapy: Clinical first-in-human study of AMG 820, a monoclonal anti-colony-stimulating factor 1 receptor antibody, in patients with advanced solid tumors. Clin. Cancer Res. 23, 5703–5710 (2017). 28655795 10.1158/1078-0432.CCR-16-3261

[20] B. Kruspig, T. Monteverde, S. Neidler, A. Hock, E. Kerr, C. Nixon, W. Clark, A. Hedley, S. Laing, S. B. Coffelt, J. Le Quesne, C. Dick, K. Vousden, C. P. Martins, D. J. Murphy, The ERBB network facilitates KRAS-driven lung tumorigenesis. Sci. Transl. Med. 10, eaao2565 (2018). 29925636 10.1126/scitranslmed.aao2565 PMC6881183

[21] D. R. McKenzie, E. E. Kara, C. R. Bastow, T. S. Tyllis, K. A. Fenix, C. E. Gregor, J. J. Wilson, R. Babb, J. C. Paton, A. Kallies, S. L. Nutt, A. Brüstle, M. Mack, I. Comerford, S. R. McColl, IL-17-producing γδ T cells switch migratory patterns between resting and activated states. Nat. Commun. 8, 15632 (2017). 28580944 10.1038/ncomms15632 PMC5465362

[22] A. Akitsu, H. Ishigame, S. Kakuta, S.-H. Chung, S. Ikeda, K. Shimizu, S. Kubo, Y. Liu, M. Umemura, G. Matsuzaki, Y. Yoshikai, S. Saijo, Y. Iwakura, IL-1 receptor antagonist-deficient mice develop autoimmune arthritis due to intrinsic activation of IL-17-producing CCR2^+^ Vγ6^+^ γδ T cells. Nat. Commun. 6, 7464 (2015). 26108163 10.1038/ncomms8464 PMC4521288

[23] R. R. Weng, H.-H. Lu, C.-T. Lin, C.-C. Fan, R.-S. Lin, T.-C. Huang, S.-Y. Lin, Y.-J. Huang, Y.-H. Juan, Y.-C. Wu, Z.-C. Hung, C. Liu, X.-H. Lin, W.-C. Hsieh, T.-Y. Chiu, J.-C. Liao, Y.-L. Chiu, S.-Y. Chen, C.-J. Yu, H.-C. Tsai, Epigenetic modulation of immune synaptic-cytoskeletal networks potentiates γδ T cell-mediated cytotoxicity in lung cancer. Nat. Commun. 12, 2163 (2021). 33846331 10.1038/s41467-021-22433-4 PMC8042060

[24] S. Jin, C. F. Guerrero-Juarez, L. Zhang, I. Chang, R. Ramos, C.-H. Kuan, P. Myung, M. V. Plikus, Q. Nie, Inference and analysis of cell-cell communication using CellChat. Nat. Commun. 12, 1088 (2021). 33597522 10.1038/s41467-021-21246-9 PMC7889871

[25] Y. Hao, S. Hao, E. Andersen-Nissen, W. M. Mauck, S. Zheng, A. Butler, M. J. Lee, A. J. Wilk, C. Darby, M. Zager, P. Hoffman, M. Stoeckius, E. Papalexi, E. P. Mimitou, J. Jain, A. Srivastava, T. Stuart, L. M. Fleming, B. Yeung, A. J. Rogers, J. M. McElrath, C. A. Blish, R. Gottardo, P. Smibert, R. Satija, Integrated analysis of multimodal single-cell data. Cell 184, 3573–3587.e29 (2021). 34062119 10.1016/j.cell.2021.04.048 PMC8238499

[26] G. Pizzolato, H. Kaminski, M. Tosolini, D. M. Franchini, F. Pont, F. Martins, C. Valle, D. Labourdette, S. Cadot, A. Quillet-Mary, M. Poupot, C. Laurent, L. Ysebaert, S. Meraviglia, F. Dieli, P. Merville, P. Milpied, J. Déchanet-Merville, J. J. Fournié, Single-cell RNA sequencing unveils the shared and the distinct cytotoxic hallmarks of human TCRVδ1 and TCRVδ2 γδ T lymphocytes. Proc. Natl. Acad. Sci. U.S.A. 116, 11906–11915 (2019). 31118283 10.1073/pnas.1818488116 PMC6576116

[27] T. T. Tapmeier, J. H. Howell, L. Zhao, B. W. Papiez, J. A. Schnabel, R. J. Muschel, A. Gal, Evolving polarisation of infiltrating and alveolar macrophages in the lung during metastatic progression of melanoma suggests CCR1 as a therapeutic target. Oncogene 41, 5032–5045 (2022). 36241867 10.1038/s41388-022-02488-3 PMC9652148

[28] L. Medina-Ruiz, R. Bartolini, G. J. Wilson, D. P. Dyer, F. Vidler, C. E. Hughes, F. Schuette, S. Love, M. Pingen, A. J. Hayes, J. Fu, A. F. Stewart, G. J. Graham, Analysis of combinatorial chemokine receptor expression dynamics using multi-receptor reporter mice. Elife 11, e72418 (2022). 35699420 10.7554/eLife.72418 PMC9236609

[29] J. McCowan, F. Fercoq, P. M. Kirkwood, W. T’Jonck, L. M. Hegarty, C. M. Mawer, R. Cunningham, A. S. Mirchandani, A. Hoy, D. C. Humphries, G. R. Jones, C. G. Hansen, N. Hirani, S. J. Jenkins, S. Henri, B. Malissen, S. R. Walmsley, D. H. Dockrell, P. T. K. Saunders, L. M. Carlin, C. C. Bain, The transcription factor EGR2 is indispensable for tissue-specific imprinting of alveolar macrophages in health and tissue repair. Sci. Immunol. 6, 2132 (2021). 10.1126/sciimmunol.abj2132 PMC7612216 34797692

[30] N. Joshi, S. Watanabe, R. Verma, R. P. Jablonski, C.-I. Chen, P. Cheresh, N. S. Markov, P. A. Reyfman, A. C. Mcquattie-Pimentel, L. Sichizya, Z. Lu, R. Piseaux-Aillon, D. Kirchenbuechler, A. S. Flozak, C. J. Gottardi, C. M. Cuda, H. Perlman, M. Jain, D. W. Kamp, G. R. Scott Budinger, A. V. Misharin, A spatially restricted fibrotic niche in pulmonary fibrosis is sustained by M-CSF/M-CSFR signalling in monocyte-derived alveolar macrophages. Eur. Respir. J. 55, 1900646 (2020). 31601718 10.1183/13993003.00646-2019 PMC6962769

[31] K. Street, D. Risso, R. B. Fletcher, D. Das, J. Ngai, N. Yosef, E. Purdom, S. Dudoit, Slingshot: Cell lineage and pseudotime inference for single-cell transcriptomics. BMC Genomics 19, 1–16 (2018). 29914354 10.1186/s12864-018-4772-0 PMC6007078

[32] Z. Tang, B. Kang, C. Li, T. Chen, Z. Zhang, GEPIA2: An enhanced web server for large-scale expression profiling and interactive analysis. Nucleic Acids Res. 47, W556–W560 (2019). 31114875 10.1093/nar/gkz430 PMC6602440

[33] P.-L. Loyher, P. Hamon, M. Laviron, A. Meghraoui-Kheddar, E. Goncalves, Z. Deng, S. Torstensson, N. Bercovici, C. Baudesson de Chanville, B. Combadière, F. Geissmann, A. Savina, C. Combadière, A. Boissonnas, Macrophages of distinct origins contribute to tumor development in the lung. J. Exp. Med. 215, 2536–2553 (2018). 30201786 10.1084/jem.20180534 PMC6170177

[34] L. B. Mostaço-Guidolin, A. C.-T. Ko, F. Wang, B. Xiang, M. Hewko, G. Tian, A. Major, M. Shiomi, M. G. Sowa, Collagen morphology and texture analysis: From statistics to classification. Sci. Rep. 3, 2190 (2013). 23846580 10.1038/srep02190 PMC3709165

[35] R. J. Hewitt, F. Puttur, D. C. A. Gaboriau, F. Fercoq, M. Fresquet, W. J. Traves, L. L. Yates, S. A. Walker, P. L. Molyneaux, S. V. Kemp, A. G. Nicholson, A. Rice, E. Roberts, R. Lennon, L. M. Carlin, A. J. Byrne, T. M. Maher, C. M. Lloyd, Lung extracellular matrix modulates KRT5^+^ basal cell activity in pulmonary fibrosis. Nat. Commun. 14, 6039 (2023). 37758700 10.1038/s41467-023-41621-y PMC10533905

[36] M. R. Mamedov, A. Scholzen, R. V. Nair, K. Cumnock, J. A. Kenkel, J. H. M. Oliveira, D. L. Trujillo, N. Saligrama, Y. Zhang, F. Rubelt, D. S. Schneider, Y.-H. Chien, R. W. Sauerwein, M. M. Davis, A macrophage colony-stimulating-factor-producing γδ T cell subset prevents malarial parasitemic recurrence. Immunity 48, 350–363.e7 (2018). 29426701 10.1016/j.immuni.2018.01.009 PMC5956914

[37] M. Chulanetra, W. Chaicumpa, Revisiting the mechanisms of immune evasion employed by human parasites. Front. Cell. Infect. Microbiol. 11, 702125 (2021). 34395313 10.3389/fcimb.2021.702125 PMC8358743

[38] S. Rashidi, C. Fernández-Rubio, R. Manzano-Román, R. Mansouri, R. Shafiei, M. Ali-Hassanzadeh, A. Barazesh, M. Karimazar, G. Hatam, P. Nguewa, Potential therapeutic targets shared between leishmaniasis and cancer. Parasitology 148, 655–671 (2021). 33536086 10.1017/S0031182021000160 PMC10090780

[39] P. Babu Narasimhan, L. Akabas, S. Tariq, N. Huda, S. Bennuru, H. Sabzevari, R. Hofmeister, T. B. Nutman, R. Tolouei Semnani, Similarities and differences between helminth parasites and cancer cell lines in shaping human monocytes: Insights into parallel mechanisms of immune evasion. PLOS Negl. Trop. Dis. 12, e0006404 (2018). 29668679 10.1371/journal.pntd.0006404 PMC5927465

[40] F. Puttur, L. G. Gregory, C. M. Lloyd, Airway macrophages as the guardians of tissue repair in the lung. Immunol. Cell Biol. 97, 246–257 (2019). 30768869 10.1111/imcb.12235

[41] R. Wiesheu, S. B. Coffelt, From backstage to the spotlight: γδT cells in cancer. Cancer Cell 42, 1637–1642 (2024). 39270647 10.1016/j.ccell.2024.08.017

[42] I. Sandrock, A. Reinhardt, S. Ravens, C. Binz, A. Wilharm, J. Martins, L. Oberdörfer, L. Tan, S. Lienenklaus, B. Zhang, R. Naumann, Y. Zhuang, A. Krueger, R. Förster, I. Prinz, Genetic models reveal origin, persistence and nonredundant functions of IL-17-producing γδ T cells. J. Exp. Med. 215, 3006–3018 (2018). 30455268 10.1084/jem.20181439 PMC6279411

[43] C. Xu, S. Li, T. S. Fulford, S. N. Christo, L. K. Mackay, D. H. Gray, A. P. Uldrich, D. G. Pellicci, D. I. Godfrey, H. F. Koay, Expansion of MAIT cells in the combined absence of NKT and γδ-T cells. Mucosal Immunol. 16, 446–461 (2023). 37182737 10.1016/j.mucimm.2023.05.003

[44] B. S. Reis, P. W. Darcy, I. Z. Khan, C. S. Moon, A. E. Kornberg, V. S. Schneider, Y. Alvarez, O. Eleso, C. Zhu, M. Schernthanner, A. Lockhart, A. Reed, J. Bortolatto, T. B. R. Castro, A. M. Bilate, S. Grivennikov, A. S. Han, D. Mucida, TCR-Vgd usage distinguishes protumor from antitumor intestinal gd T cell subsets. Science 377, 276–284 (2022). 35857588 10.1126/science.abj8695 PMC9326786

[45] B. S. Sheridan, P. A. Romagnoli, Q. M. Pham, H. H. Fu, F. Alonzo, W. D. Schubert, N. E. Freitag, L. Lefrançois, γδ T cells exhibit multifunctional and protective memory in intestinal tissues. Immunity 39, 184–195 (2013). 23890071 10.1016/j.immuni.2013.06.015 PMC3749916

[46] C. Koenecke, V. Chennupati, S. Schmitz, B. Malissen, R. Förster, I. Prinz, In vivo application of mAb directed against the γδ TCR does not deplete but generates “invisible” γδ T cells. Eur. J. Immunol. 39, 372–379 (2009). 19130484 10.1002/eji.200838741

[47] C. Harmon, A. Zaborowski, H. Moore, P. St. Louis, K. Slattery, D. Duquette, J. Scanlan, H. Kane, B. Kunkemoeller, C. L. McIntyre, A. N. Scannail, B. Moran, A. C. Anderson, D. Winter, D. Brennan, M. A. Brehm, L. Lynch, γδ T cell dichotomy with opposing cytotoxic and wound healing functions in human solid tumors. Nat. Cancer 4, 1122–1137 (2023). 37474835 10.1038/s43018-023-00589-w

[48] S. C. Casey, V. Baylot, D. W. Felsher, The MYC oncogene is a global regulator of the immune response. Blood 131, 2007–2015 (2018). 29514782 10.1182/blood-2017-11-742577 PMC5934797

[49] M. Kalkat, J. De Melo, K. A. Hickman, C. Lourenco, C. Redel, D. Resetca, A. Tamachi, W. B. Tu, L. Z. Penn, MYC deregulation in primary human cancers. *Genes* (*Basel*) 8, 151 (2017). 28587062 10.3390/genes8060151 PMC5485515

[50] R. M. Kortlever, N. M. Sodir, C. H. Wilson, D. L. Burkhart, L. Pellegrinet, L. Brown Swigart, T. D. Littlewood, G. I. Evan, Myc cooperates with Ras by programming inflammation and immune suppression. Cell 171, 1301–1315.e14 (2017). 29195074 10.1016/j.cell.2017.11.013 PMC5720393

[51] E. Mugarza, F. van Maldegem, J. Boumelha, C. Moore, S. Rana, M. Llorian Sopena, P. East, R. Ambler, P. Anastasiou, P. Romero-Clavijo, K. Valand, M. Cole, M. Molina-Arcas, J. Downward, Therapeutic KRAS G12C inhibition drives effective interferon-mediated antitumor immunity in immunogenic lung cancers. Sci. Adv. 8, 8780 (2022). 10.1126/sciadv.abm8780 PMC9299537 35857848

[52] G. Clark, H. Donninger, S. Piscuoglio, A. R. Halvorsen, L. Silwal-Pandit, L. A. Meza-Zepeda, D. Vodak, P. Vu, C. Sagerup, E. Hovig, O. Myklebost, A.-L. Børresen-Dale, O. T. Brustugun, Å. Helland, TP53 mutation spectrum in smokers and never smoking lung cancer patients. Front. Genet. 7, 85 (2016). 27242894 10.3389/fgene.2016.00085 PMC4863128

[53] N. Jarzebska, E. S. Karetnikova, A. G. Markov, M. Kasper, R. N. Rodionov, P. M. Spieth, Scarred lung. An update on radiation-induced pulmonary fibrosis. Front. Med. 7, 585756 (2021). 10.3389/fmed.2020.585756 PMC7843914 33521012

[54] A. J. Patel, P. Nightingale, B. Naidu, M. T. Drayson, G. W. Middleton, A. Richter, Characterising the impact of pneumonia on outcome in non-small cell lung cancer: Identifying preventative strategies. J. Thorac. Dis. 12, 2236 (2020). 32642129 10.21037/jtd.2020.04.49 PMC7330320

[55] E. L. Jackson, N. Willis, K. Mercer, R. T. Bronson, D. Crowley, R. Montoya, T. Jacks, D. A. Tuveson, Analysis of lung tumor initiation and progression using conditional expression of oncogenic K-Ras. Genes Dev. 15, 3243–3248 (2001). 11751630 10.1101/gad.943001 PMC312845

[56] S. Itohara, P. Mombaerts, J. Lafaille, J. Iacomini, A. Nelson, A. R. Clarke, M. L. Hooper, A. Farr, S. Tonegawa, T cell receptor δ gene mutant mice: Independent generation of αβ T cells and programmed rearrangements of γδ TCR genes. Cell 72, 337–348 (1993). 8381716 10.1016/0092-8674(93)90112-4

[57] S. Sunaga, K. Maki, Y. Komagata, K. Ikuta, J.-I. Miyazaki, Efficient removal ofloxP-flanked DNA sequences in a gene-targeted locus by transient expression of Cre recombinase in fertilized eggs. Mol. Reprod. Dev. 46, 109–113 (1997). 9021742 10.1002/(SICI)1098-2795(199702)46:2<109::AID-MRD1>3.0.CO;2-U

[58] D. J. Murphy, M. R. Junttila, L. Pouyet, A. Karnezis, K. Shchors, D. A. Bui, L. Brown-Swigart, L. Johnson, G. I. Evan, Distinct thresholds govern Myc’s biological output in vivo. Cancer Cell 14, 447–457 (2008). 19061836 10.1016/j.ccr.2008.10.018 PMC2723751

[59] S. Andrews, FastQC: A quality control tool for high throughput sequence data (2010); https://www.bioinformatics.babraham.ac.uk/projects/fastqc/.

[60] S. Chen, Y. Zhou, Y. Chen, J. Gu, fastp: An ultra-fast all-in-one FASTQ preprocessor. Bioinformatics 34, i884–i890 (2018). 30423086 10.1093/bioinformatics/bty560 PMC6129281

[61] S. W. Wingett, S. Andrews, FastQ screen: A tool for multi-genome mapping and quality control. F1000Res 7, 1338 (2018). 30254741 10.12688/f1000research.15931.1 PMC6124377

[62] K. Nikolatou, E. Sandilands, A. Román-Fernández, E. M. Cumming, E. Freckmann, S. Lilla, L. Buetow, L. McGarry, M. Neilson, R. Shaw, D. Strachan, C. Miller, D. T. Huang, I. A. McNeish, J. C. Norman, S. Zanivan, D. M. Bryant, PTEN deficiency exposes a requirement for an ARF GTPase module for integrin-dependent invasion in ovarian cancer. EMBO J. 42, e113987 (2023). 37577760 10.15252/embj.2023113987 PMC10505920

[63] F. Cunningham, P. Achuthan, W. Akanni, J. Allen, M. R. Amode, I. M. Armean, R. Bennett, J. Bhai, K. Billis, S. Boddu, C. Cummins, C. Davidson, J. Dodiya, A. Gall, C. García Girón, G. Girón, L. Gil, T. Grego, L. Haggerty, E. Haskell, T. Hourlier, O. G. Izuogu, S. H. Janacek, T. Juettemann, M. Kay, M. R. Laird, I. Lavidas, Z. Liu, J. E. Loveland, J. C. Marugán, T. Maurel, A. C. McMahon, B. Moore, J. Morales, J. M. Mudge, M. Nuhn, D. Ogeh, A. Parker, A. Parton, M. Patricio, A. Imran, A. Salam, B. M. Schmitt, H. Schuilenburg, D. Sheppard, H. Sparrow, E. Stapleton, M. Szuba, K. Taylor, G. Threadgold, A. Thormann, A. Vullo, B. Walts, A. Winterbottom, A. Zadissa, M. Chakiachvili, A. Frankish, S. E. Hunt, M. Kostadima, N. Langridge, F. J. Martin, M. Muffato, E. Perry, M. Ruffier, D. M. Staines, S. J. Trevanion, B. L. Aken, A. D. Yates, D. R. Zerbino, P. Flicek, Ensembl 2019. Nucleic Acids Res. 47, 745–751 (2018). 10.1093/nar/gky1113 PMC6323964 30407521

[64] R. A. Amezquita, A. T. L. Lun, E. Becht, V. J. Carey, L. N. Carpp, L. Geistlinger, F. Marini, K. Rue-Albrecht, D. Risso, C. Soneson, L. Waldron, H. Pagès, M. L. Smith, W. Huber, M. Morgan, R. Gottardo, S. C. Hicks, Data infrastructure orchestrating single-cell analysis with Bioconductor. Nat. Methods 17, 137–145 (2020). 31792435 10.1038/s41592-019-0654-x PMC7358058

[65] R. Browaeys, W. Saelens, Y. Saeys, NicheNet: Modeling intercellular communication by linking ligands to target genes. Nat. Methods 17, 159–162 (2020). 31819264 10.1038/s41592-019-0667-5

[66] D. A. Barbie, P. Tamayo, J. S. Boehm, S. Y. Kim, S. E. Moody, I. F. Dunn, A. C. Schinzel, P. Sandy, E. Meylan, C. Scholl, S. Fröhling, E. M. Chan, M. L. Sos, K. Michel, C. Mermel, S. J. Silver, B. A. Weir, J. H. Reiling, Q. Sheng, P. B. Gupta, R. C. Wadlow, H. Le, S. Hoersch, B. S. Wittner, S. Ramaswamy, D. M. Livingston, D. M. Sabatini, M. Meyerson, R. K. Thomas, E. S. Lander, J. P. Mesirov, D. E. Root, D. G. Gilliland, T. Jacks, W. C. Hahn, Systematic RNA interference reveals that oncogenic KRAS-driven cancers require TBK1. Nature 462, 108–112 (2009). 19847166 10.1038/nature08460 PMC2783335

[67] A. Subramanian, P. Tamayo, V. K. Mootha, S. Mukherjee, B. L. Ebert, M. A. Gillette, A. Paulovich, S. L. Pomeroy, T. R. Golub, E. S. Lander, J. P. Mesirov, Gene set enrichment analysis: A knowledge-based approach for interpreting genome-wide expression profiles. Proc. Natl. Acad. Sci. U.S.A. 102, 15545–15550 (2005). 16199517 10.1073/pnas.0506580102 PMC1239896

[68] J. Griss, G. Viteri, K. Sidiropoulos, V. Nguyen, A. Fabregat, H. Hermjakob, ReactomeGSA-efficient multi-omics comparative pathway analysis. Mol. Cell. Proteomics 19, 2115–2124 (2020). 32907876 10.1074/mcp.TIR120.002155 PMC7710148

[69] S. Hänzelmann, R. Castelo, J. Guinney, GSVA: Gene set variation analysis for microarray and RNA-seq data. BMC Bioinformatics 14, 7 (2013). 23323831 10.1186/1471-2105-14-7 PMC3618321

[70] M. Gillespie, B. Jassal, R. Stephan, M. Milacic, K. Rothfels, A. Senff-Ribeiro, J. Griss, C. Sevilla, L. Matthews, C. Gong, C. Deng, T. Varusai, E. Ragueneau, Y. Haider, B. May, V. Shamovsky, J. Weiser, T. Brunson, N. Sanati, L. Beckman, X. Shao, A. Fabregat, K. Sidiropoulos, J. Murillo, G. Viteri, J. Cook, S. Shorser, G. Bader, E. Demir, C. Sander, R. Haw, G. Wu, L. Stein, H. Hermjakob, P. D’eustachio, The reactome pathway knowledgebase 2022. Nucleic Acids Res. 50, D687–D692 (2022). 34788843 10.1093/nar/gkab1028 PMC8689983

[71] P. Ewels, M. Ns Magnusson, S. Lundin, M. K. Aller, MultiQC: Summarize analysis results for multiple tools and samples in a single report. Bioinformatics 32, 3047–3048 (2016). 27312411 10.1093/bioinformatics/btw354 PMC5039924

[72] T. Kluyver, B. Ragan-Kelley, F. Pérez, B. Granger, M. Bussonnier, J. Frederic, K. Kelley, J. Hamrick, J. Grout, S. Corlay, P. Ivanov, D. Avila, S. Abdalla, C. Willing, Jupyter Development Team, “Jupyter Notebooks—A publishing format for reproducible computational workflows” in *Positioning and Power in Academic Publishing: Players, Agents and Agendas* (IOS Press, 2016), pp. 87–90.

[73] Posit Team, RStudio: Integrated Development Environment for R. (2025); http://posit.co/.

[74] F. J. Martin, M. R. Amode, A. Aneja, O. Austine-Orimoloye, A. G. Azov, I. Barnes, A. Becker, R. Bennett, A. Berry, J. Bhai, S. K. Bhurji, A. Bignell, S. Boddu, P. R. Branco Lins, L. Brooks, S. B. Ramaraju, M. Charkhchi, A. Cockburn, L. Da Rin Fiorretto, C. Davidson, K. Dodiya, S. Donaldson, B. El Houdaigui, T. El Naboulsi, R. Fatima, C. G. Giron, T. Genez, G. S. Ghattaoraya, J. G. Martinez, C. Guijarro, M. Hardy, Z. Hollis, T. Hourlier, T. Hunt, M. Kay, V. Kaykala, T. Le, D. Lemos, D. Marques-Coelho, J. C. Marugán, G. A. Merino, L. P. Mirabueno, A. Mushtaq, S. N. Hossain, D. N. Ogeh, M. P. Sakthivel, A. Parker, M. Perry, I. Piližota, I. Prosovetskaia, J. G. Pérez-Silva, A. I. A. Salam, N. Saraiva-Agostinho, H. Schuilenburg, D. Sheppard, S. Sinha, B. Sipos, W. Stark, E. Steed, R. Sukumaran, D. Sumathipala, M.-M. Suner, L. Surapaneni, K. Sutinen, M. Szpak, F. F. Tricomi, D. Urbina-Gómez, A. Veidenberg, T. A. Walsh, B. Walts, E. Wass, N. Willhoft, J. Allen, J. Alvarez-Jarreta, M. Chakiachvili, B. Flint, S. Giorgetti, L. Haggerty, G. R. Ilsley, J. E. Loveland, B. Moore, J. M. Mudge, J. Tate, D. Thybert, S. J. Trevanion, A. Winterbottom, A. Frankish, S. E. Hunt, M. Ruffier, F. Cunningham, S. Dyer, R. D. Finn, K. L. Howe, P. W. Harrison, A. D. Yates, P. Flicek, Ensembl 2023. Nucleic Acids Res. 51, D933–D941 (2023). 36318249 10.1093/nar/gkac958 PMC9825606

[75] A. Dobin, C. A. Davis, F. Schlesinger, J. Drenkow, C. Zaleski, S. Jha, P. Batut, M. Chaisson, T. R. Gingeras, STAR: Ultrafast universal RNA-seq aligner. Bioinformatics 29, 15–21 (2013). 23104886 10.1093/bioinformatics/bts635 PMC3530905

[76] Y. Liao, G. K. Smyth, W. Shi, featureCounts: An efficient general purpose program for assigning sequence reads to genomic features. Bioinformatics 30, 923–930 (2014). 24227677 10.1093/bioinformatics/btt656

[77] R Core Team, R: A language and environment for statistical computing, R Foundation for Statistical Computing (2020); https://R-project.org/.

[78] W. Huber, V. J. Carey, R. Gentleman, S. Anders, M. Carlson, B. S. Carvalho, H. C. Bravo, S. Davis, L. Gatto, T. Girke, R. Gottardo, F. Hahne, K. D. Hansen, R. A. Irizarry, M. Lawrence, M. I. Love, J. MacDonald, V. Obenchain, A. K. Oleś, H. Pagès, A. Reyes, P. Shannon, G. K. Smyth, D. Tenenbaum, L. Waldron, M. Morgan, Orchestrating high-throughput genomic analysis with Bioconductor. Nat. Methods 12, 115–121 (2015). 25633503 10.1038/nmeth.3252 PMC4509590

[79] M. I. Love, W. Huber, S. Anders, Moderated estimation of fold change and dispersion for RNA-seq data with DESeq2. Genome Biol. 15, 550 (2014). 25516281 10.1186/s13059-014-0550-8 PMC4302049

[80] A. Zhu, J. G. Ibrahim, M. I. Love, Heavy-tailed prior distributions for sequence count data: Removing the noise and preserving large differences. Bioinformatics 35, 2084–2092 (2019). 30395178 10.1093/bioinformatics/bty895 PMC6581436

[81] U. Raudvere, L. Kolberg, I. Kuzmin, T. Arak, P. Adler, H. Peterson, J. Vilo, g:Profiler: A web server for functional enrichment analysis and conversions of gene lists (2019 update). Nucleic Acids Res. 47, W191–W198 (2019). 31066453 10.1093/nar/gkz369 PMC6602461

[82] S. Sayols, rrvgo: A Bioconductor package for interpreting lists of Gene Ontology terms. MicroPubl. Biol. 2023, 10.17912/micropub.biology.000811 (2023). 10.17912/micropub.biology.000811 PMC10155054 37151216

[83] P. Bankhead, M. B. Loughrey, J. A. Fernández, Y. Dombrowski, D. G. Mcart, P. D. Dunne, S. Mcquaid, R. T. Gray, L. J. Murray, H. G. Coleman, J. A. James, M. Salto-Tellez, P. W. Hamilton, QuPath: Open source software for digital pathology image analysis. Sci. Rep. 7, 16878 (2017). 29203879 10.1038/s41598-017-17204-5 PMC5715110

[84] J. Schindelin, I. Arganda-Carreras, E. Frise, V. Kaynig, M. Longair, T. Pietzsch, S. Preibisch, C. Rueden, S. Saalfeld, B. Schmid, J. Y. Tinevez, D. J. White, V. Hartenstein, K. Eliceiri, P. Tomancak, A. Cardona, Fiji: An open-source platform for biological-image analysis. Nat. Methods 9, 676–682 (2012). 22743772 10.1038/nmeth.2019 PMC3855844

[85] S. Van Der Walt, J. L. Schönberger, J. Nunez-Iglesias, F. Boulogne, J. D. Warner, N. Yager, E. Gouillart, T. Yu, Scikit-image: Image processing in Python. PeerJ 2, e453 (2014). 25024921 10.7717/peerj.453 PMC4081273

[86] A. Parslow, A. Cardona, R. J. Bryson-Richardson, Sample drift correction following 4D confocal time-lapse imaging. J. Vis. Exp. 86, 51086 (2014). 10.3791/51086 PMC4166950 24747942

[87] M. Pachitariu, C. Stringer, Cellpose 2.0: How to train your own model. Nat. Methods 19, 1634–1641 (2022). 36344832 10.1038/s41592-022-01663-4 PMC9718665

[88] K. Ulicna, G. Vallardi, G. Charras, A. R. Lowe, Automated deep lineage tree analysis using a Bayesian single cell tracking approach. Front. Comput. Sci. 3, 734559 (2021).

