# Supplementary material for: Vγ1 γδ T cells steer airway macrophages toward a profibrotic response in an autochthonous lung cancer mouse model
Source: Sci Adv. 2026 Mar 4;12(10):eadu8802. doi: 10.1126/sciadv.adu8802 (PMC12959402; doi:10.1126/sciadv.adu8802)
Supplement: Supplementary file 1 — Figs. S1 to S4 Legends for movies S1 to S7 [file sciadv.adu8802_sm.pdf]

Science Advances
AAAS

Supplementary Materials for

Vγ1 γδ T cells steer airway macrophages toward a profibrotic response in an autochthonous lung cancer mouse model

Ximena L. Raffo-Iraolagoitia *et al.*

Corresponding author: Ximena L. Raffo-Iraolagoitia, x.raffo@crukscotlandinstitute.ac.uk;
Leo M. Carlin, leo.carlin@glasgow.ac.uk

Sci. Adv. **12**, eadu8802 (2026)
DOI: 10.1126/sciadv.adu8802

The PDF file includes:

Figs. S1 to S4
Legends for movies S1 to S7

Other Supplementary Material for this manuscript includes the following:

Movies S1 to S7

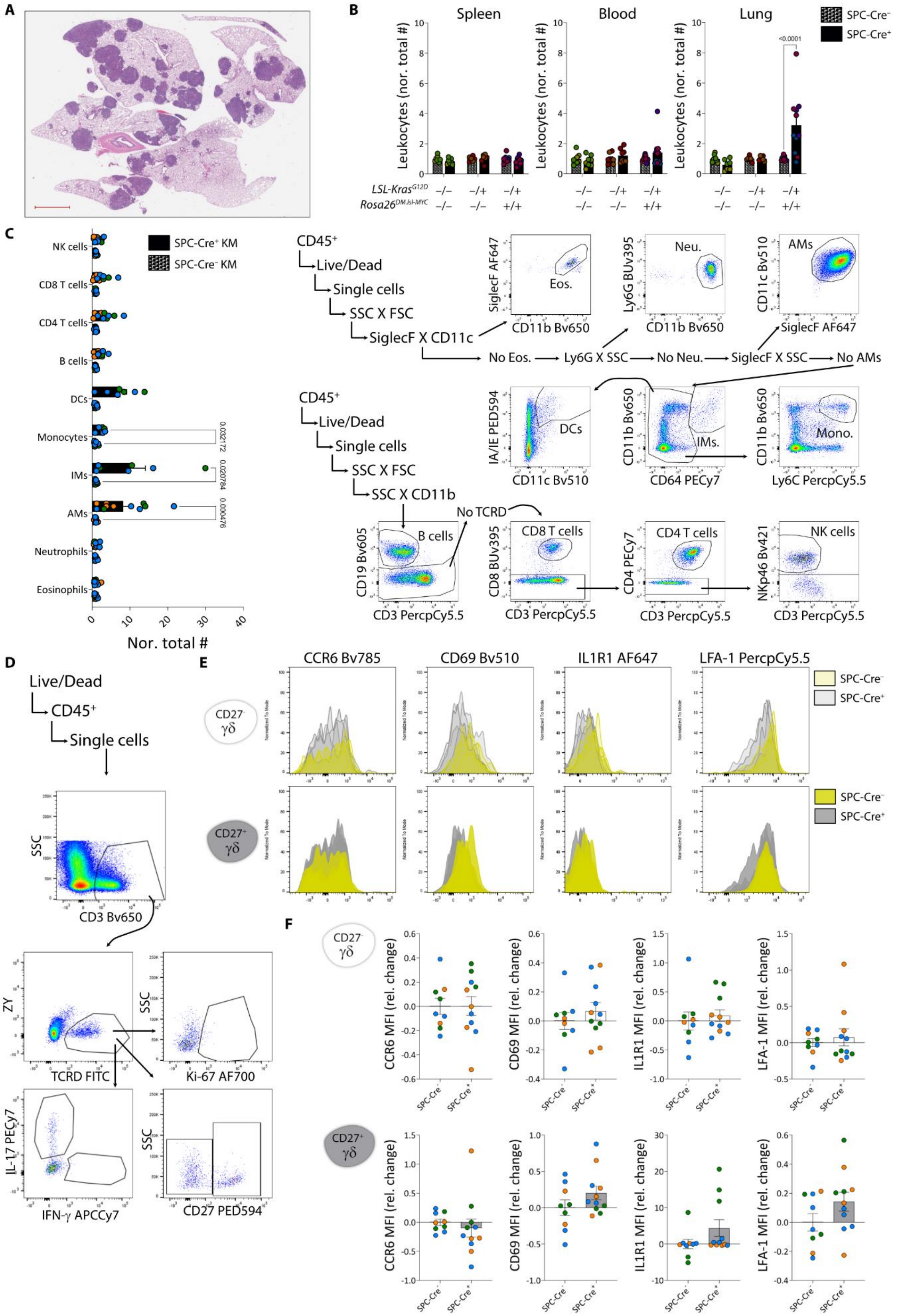
A
B
Spleen
Blood
Lung
SPC-Cre⁻
SPC-Cre⁺
<0.0001
Leukocytes (nor. total #)
LSL-Kras G12D
Rosa26 DM.lsl-MYC
–/– –/+ –/+
–/– –/– +/+
C
SPC-Cre⁺ KM
SPC-Cre⁻ KM
NK cells
CD8 T cells
CD4 T cells
B cells
DCs
Monocytes
IMs
AMs
Neutrophils
Eosinophils
0.032172
0.020784
0.000476
Nor. total #
CD45⁺
Live/Dead
Single cells
SSC X FSC
SiglecF X CD11c
SiglecF AF647
CD11b Bv650
Eos.
Ly6G BUv395
Neu.
CD11c Bv510
AMs
No Eos.
Ly6G X SSC
No Neu.
SiglecF X SSC
No AMs
SSC X CD11b
IA/IE PED594
DCs
CD11c Bv510
CD11b Bv650
IMs.
CD64 PECy7
Mono.
Ly6C PercpCy5.5
No TCRD
CD19 Bv605
B cells
CD3 PercpCy5.5
CD8 BUv395
CD8 T cells
CD4 PECy7
CD4 T cells
NKp46 Bv421
NK cells
D
Live/Dead
CD45⁺
Single cells
SSC
CD3 Bv650
ZY
TCRD FITC
Ki-67 AF700
IL-17 PECy7
IFN-γ APCCy7
CD27 PED594
E
CCR6 Bv785
CD69 Bv510
IL1R1 AF647
LFA-1 PercpCy5.5
CD27⁻ γδ
CD27⁺ γδ
Normalized To Mode
F
CCR6 MFI (rel. change)
CD69 MFI (rel. change)
IL1R1 MFI (rel. change)
LFA-1 MFI (rel. change)
SPC-Cre⁻
SPC-Cre⁺

Fig. S1.

Immune phenotype of KM mice. (A) Representative H&E of SPC-Cre$^+$ KM mouse lungs 8 weeks after allele activation. Scale bar: 2 mm. **(B)** Normalized total number of leukocytes in spleen, blood, and lungs for different genotypes (n = 8 mice/group; 6-7 mice/group; 9-11 mice/group). Data were analyzed by two-way ANOVA followed by Sidak's post-test. **(C)** Main leukocyte populations in lungs of KM mice (SPC-Cre$^-$ and SPC-Cre$^+$, n = 9-11 mice/group). Data were analyzed by Mann-Whitney test with Holm-Sidak correction for multiple comparisons. Schematic gating strategy on the right. **(D)** Schematic gating strategy for γδ T cells. **(E)** Histograms of gated pulmonary CD27$^-$ and CD27$^+$ γδ T cells for CCR6, CD69, IL1R1, and LFA-1, from one representative experiment in SPC-Cre$^-$ KM and SPC-Cre$^+$ KM mice (related to **Fig. 1C**). **(F)** Gated on blood CD27$^-$ (white bars) and CD27$^+$ (grey bars) γδ T cells, MFI relative change for CCR6, CD69, IL1R1, and LFA-1 in SPC-Cre$^-$ KM and SPC-Cre$^+$ KM mice (n = 9-11 mice/group). **(B, C, F)** Each dot represents a mouse, colored by independent experiment. Data are presented as mean ± SEM.

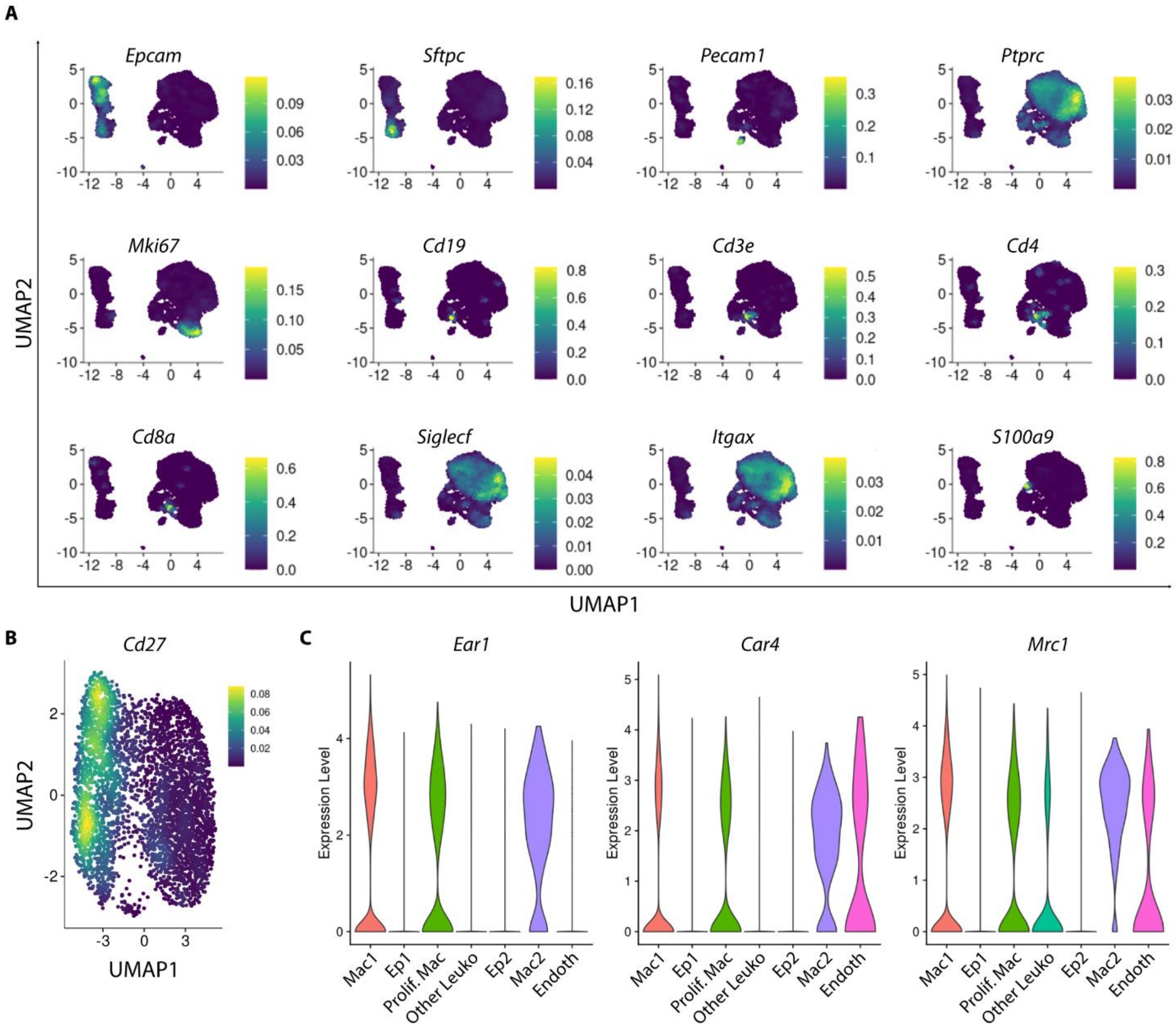

Fig. S2.

Genes used to annotate scRNA-seq clusters. (A) Density plots showing expression of *Epcam*, *Sftpc*, *Pecam1*, *Ptprc*, *Mki67*, *Cd19*, *Cd3e*, *Cd4*, *Cd8a*, *Siglecf*, *Itgax*, and *S100a9* on UMAP dimensionality reduction for scRNA-seq data from microdissected SPC-Cre^{+} KM lung tumors. **(B)** Density plot showing expression of *Cd27* on UMAP dimensionality reduction for scRNA-seq data from naïve pulmonary γδ T cells (*4*). **(C)** Violin plots showing the expression of *Ear1*, *Car4*, and *Mrc1* by cluster in the scRNA-seq data from microdissected SPC-Cre^{+} KM lung tumors.

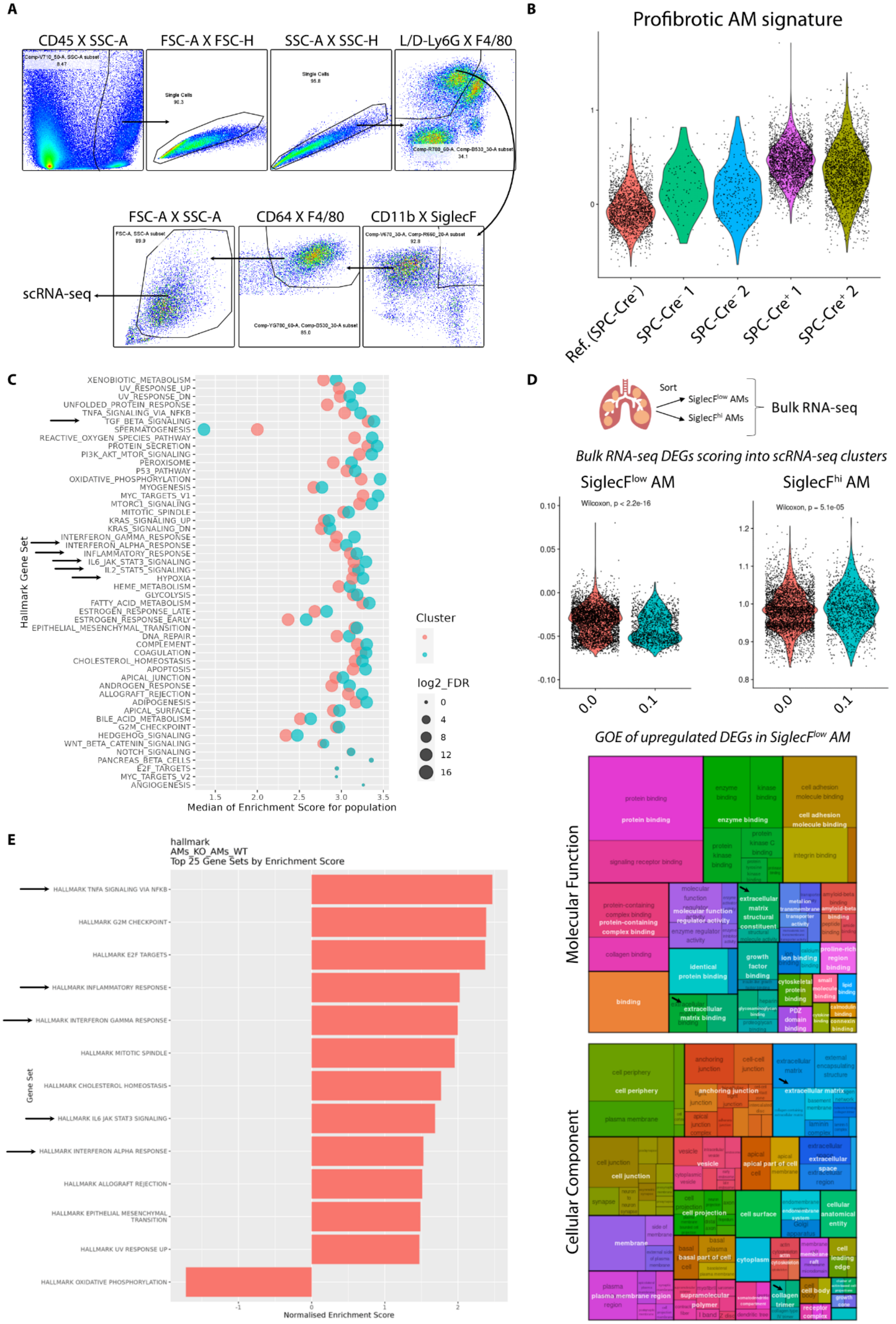
A
CD45 X SSC-A
FSC-A X FSC-H
SSC-A X SSC-H
L/D-Ly6G X F4/80
Single Cells
90.3
Single Cells
95.8
FSC-A X SSC-A
CD64 X F4/80
CD11b X SiglecF
scRNA-seq
B
Profibrotic AM signature
Ref. (SPC-Cre⁻)
SPC-Cre⁻ 1
SPC-Cre⁻ 2
SPC-Cre⁺ 1
SPC-Cre⁺ 2
C
Hallmark Gene Set
XENOBIOTIC_METABOLISM
UV_RESPONSE_UP
UV_RESPONSE_DN
UNFOLDED_PROTEIN_RESPONSE
TNFA_SIGNALING_VIA_NFKB
TGF_BETA_SIGNALING
SPERMATOGENESIS
REACTIVE_OXYGEN_SPECIES_PATHWAY
PROTEIN_SECRETION
PI3K_AKT_MTOR_SIGNALING
PEROXISOME
P53_PATHWAY
OXIDATIVE_PHOSPHORYLATION
MYOGENESIS
MYC_TARGETS_V1
MTORC1_SIGNALING
MITOTIC_SPINDLE
KRAS_SIGNALING_UP
KRAS_SIGNALING_DN
INTERFERON_GAMMA_RESPONSE
INTERFERON_ALPHA_RESPONSE
INFLAMMATORY_RESPONSE
IL6_JAK_STAT3_SIGNALING
IL2_STAT5_SIGNALING
HYPOXIA
HEME_METABOLISM
GLYCOLYSIS
FATTY_ACID_METABOLISM
ESTROGEN_RESPONSE_LATE
ESTROGEN_RESPONSE_EARLY
EPITHELIAL_MESENCHYMAL_TRANSITION
DNA_REPAIR
COMPLEMENT
COAGULATION
CHOLESTEROL_HOMEOSTASIS
APOPTOSIS
APICAL_JUNCTION
ANDROGEN_RESPONSE
ALLOGRAFT_REJECTION
ADIPOGENESIS
APICAL_SURFACE
BILE_ACID_METABOLISM
G2M_CHECKPOINT
HEDGEHOG_SIGNALING
WNT_BETA_CATENIN_SIGNALING
NOTCH_SIGNALING
PANCREAS_BETA_CELLS
E2F_TARGETS
MYC_TARGETS_V2
ANGIOGENESIS
1.5
2.0
2.5
3.0
3.5
Median of Enrichment Score for population
Cluster
log2_FDR
0
4
8
12
16
D
Sort
SiglecF^low AMs
SiglecF^hi AMs
Bulk RNA-seq
Bulk RNA-seq DEGs scoring into scRNA-seq clusters
SiglecF^low AM
SiglecF^hi AM
Wilcoxon, p < 2.2e-16
Wilcoxon, p = 5.1e-05
GOE of upregulated DEGs in SiglecF^low AM
Molecular Function
protein binding
enzyme binding
cell adhesion molecule binding
signaling receptor binding
integrin binding
protein-containing complex binding
molecular function regulator activity
extracellular matrix structural constituent
collagen binding
identical protein binding
growth factor binding
ion binding
binding
extracellular matrix binding
Cellular Component
cell periphery
anchoring junction
extracellular matrix
plasma membrane
cell junction
vesicle
apical part of cell
extracellular space
cell projection
cell surface
membrane
basal part of cell
cytoplasm
plasma membrane region
supramolecular polymer
collagen trimer
E
hallmark
AMs_KO_AMs_WT
Top 25 Gene Sets by Enrichment Score
Gene Set
HALLMARK TNFA SIGNALING VIA NFKB
HALLMARK G2M CHECKPOINT
HALLMARK E2F TARGETS
HALLMARK INFLAMMATORY RESPONSE
HALLMARK INTERFERON GAMMA RESPONSE
HALLMARK MITOTIC SPINDLE
HALLMARK CHOLESTEROL HOMEOSTASIS
HALLMARK IL6 JAK STAT3 SIGNALING
HALLMARK INTERFERON ALPHA RESPONSE
HALLMARK ALLOGRAFT REJECTION
HALLMARK EPITHELIAL MESENCHYMAL TRANSITION
HALLMARK UV RESPONSE UP
HALLMARK OXIDATIVE PHOSPHORYLATION
-1
0
1
2
Normalised Enrichment Score

Fig. S3.

scRNA-seq of macrophages from microdissected SPC-Cre^{-} and SPC-Cre^{+} KM lungs. (A) Gating strategy used for the isolation of macrophages from microdissected SPC-Cre^{-} and SPC-Cre^{+} KM lungs for scRNA-sequencing. **(B)** Violin plot showing the expression of a consensus profibrotic AM signature (*28*) by sample. **(C)** Single sample Gene Set Enrichment Analysis (ssGSEA) performed on scRNA-Seq data to evaluate Hallmark Gene Sets for cells in Clusters 0.0 and 0.1 (blue and red, respectively). Median enrichment scores shown for each cluster (x-axis), and statistical significance (log2 FDR) of differences between the two clusters indicated by point size/radius. Mann-Whitney U test, 3698 cells in Cluster 0 and 1282 cells in Cluster 1, from two Tumor samples (related to **Fig. 3D**). **(D)** Violin plots showing Module scores per cell for Clusters 0.0 and 0.1 of TA-AMs, respectively, calculated based on a module derived from significantly changed genes identified in bulk RNA-Seq between sorted SiglecF^{low} AMs and SiglecF^{hi} AMs (n= 3; top). Molecular Function and Cellular Component tree maps for enriched gene ontology terms (GOE) based on significant genes upregulated in SiglecF^{low} AMs (bottom). **(E)** Gene set enrichment analysis of Hallmark pathways in AMs from SPC-Cre^{+} KM; *Tcrd* knockout lungs (n = 3 mice/group).

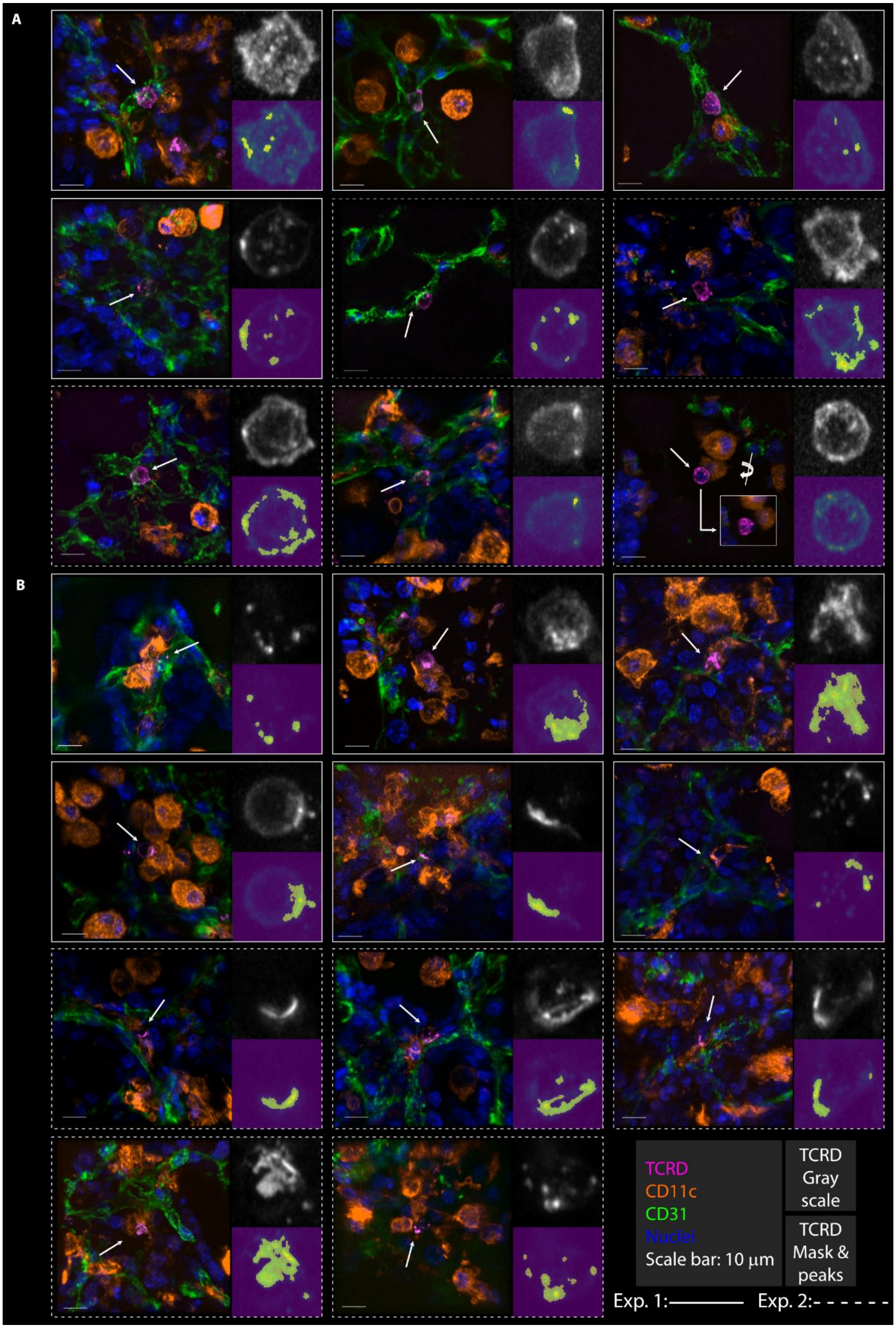
A
B
TCRD
CD11c
CD31
Nuclei
Scale bar: 10 µm
TCRD Gray scale
TCRD Mask & peaks
Exp. 1:
Exp. 2:

Fig. S4.

γδ T cells in the absence (A) or presence (B) of airway macrophage contact. Visualization of the first time point for each time-lapse. Solid and dotted outlines indicate images from two independent experiments. Left: field of view with merged channels showing vasculature (CD31, green), airway macrophages (CD11c, orange), TCRδ (magenta), and Hoechst (blue); scale bar: 10μm; white arrows indicate the γδ T cell shown in the corresponding cropped image used for analysis. Top right: region of interest (ROI) containing a single γδ T cell (TCRD, gray scale). Bottom right: TCRD masks and peaks for the same ROI.

Movie S1.

Intravascular γδ T cells. 3D view of lung parenchyma. First zoom-in shows a $CD27^-$ γδ T cell and, the second zoom-in, a $CD27^+$ γδ T cell. Vasculature (CD31, green), collagen (second harmonic generation, glow palette), leukocytes (CD45, cyan), macrophages (CD68, orange), CD27 (red), and TCRδ (magenta).

Movie S2.

Infiltrating γδ T cells. 3D view of lung tumor. Zoom-in featuring a $CD27^+$ γδ T cell within the tumor, adjacent to the vasculature. Vasculature (CD31, green), collagen (second harmonic generation, glow palette), leukocytes (CD45, cyan), macrophages (CD68, orange), CD27 (red), and TCRδ (magenta).

Movie S3.

Interacting γδ T cells. 3D view of lung tumor-associated adventitial cuff. Zoom-in featuring a $CD27^+$ γδ T cell in contact with a macrophage. Vasculature (CD31, green), collagen (second harmonic generation, glow palette), leukocytes (CD45, cyan), macrophages (CD68, orange), CD27 (red), and TCRD (magenta).

Movie S4.

Long-lasting γδ T cells interactions with airway macrophages. Time-lapse on a precision-cut lung slice of tumor margin imaged every 95.5 seconds. Zoom-ins featuring γδ T cells interacting with airway macrophages. Vasculature (CD31, green), airway macrophages (CD11c, orange), TCRδ (magenta), and Hoechst (blue).

Movie S5.

γδ T cells in the absence of airway macrophage contact. Time-lapses on precision-cut lung slices of tumor-bearing mice imaged every 35 seconds. Each field of view featuring a γδ T cell which is not in contact with airway macrophages. Vasculature (CD31, green), airway macrophages (CD11c, orange), TCRδ (magenta), and Hoechst (blue). Scale bar: 10μm. Solid and dotted outlines denote time-lapses from two independent experiments.

Movie S6.

γδ T cells in the presence of airway macrophage contact. Time-lapses on precision-cut lung slices of tumor-bearing mice imaged every 35 seconds. Each field of view featuring a γδ T cell which is in contact with airway macrophages. Vasculature (CD31, green), airway macrophages (CD11c, orange), TCRδ (magenta), and Hoechst (blue). Scale bar: 10μm. Solid and dotted outlines denote time-lapses from two independent experiments.

Movie S7.

Different interactions between γδ T cells and airway macrophages *ex vivo*. Representative raw time lapse from IncuCyte. Airway macrophages (green), Cytotox (red), and γδ T cells (phase contrast) **(top left)**. Tracking of interactions (yellow squares) in segmented images **(bottom left)**. On the right, examples of different behaviors.
